# Digital health literacy, behavior and knowledge of adolescents for hand hygiene during the COVID-19 pandemic*

**DOI:** 10.1590/1518-8345.7228.4308

**Published:** 2024-08-30

**Authors:** Rafaela Aparecida Prata, Juliana Bastoni da Silva, Sidiany Mendes Pimentel, Hélio Rubens de Carvalho Nunes, Marla Andréia Garcia de Avila

**Affiliations:** 1 Universidade Estadual Paulista, Departamento de Enfermagem, Botucatu, SP, Brazil.; 2 Universidade Federal do Tocantins, Departamento de Enfermagem, Palmas, TO, Brazil.

**Keywords:** Health Literacy, Hand Disinfection, Hand Hygiene, COVID-19, Adolescent, Adolescent Health

## Abstract

**Objective:**

to investigate factors associated with digital health literacy, hand hygiene knowledge and behavior among Brazilian adolescents during the COVID-19 pandemic.

**Method:**

cross-sectional study with 473 adolescents aged 15 to 19. Data were collected virtually between June and August 2021 through a questionnaire on sociodemographic characteristics, digital health literacy, knowledge and behavior regarding hand hygiene. Variables were adjusted using multiple linear regression models with normal response.

**Results:**

the average digital health literacy score was 29.89 ±5.30; for hand hygiene knowledge and behavior was 13.1 ±1.5 and 11.1±2.7, respectively. Higher knowledge and behavior scores for hand hygiene were associated with having completed high school, compared to those attending college, among those who attend health courses, seek information about the pandemic and in scientific articles. Higher digital health literacy scores in adolescents who did not wear masks when playing sports (p= 0.017). There was an association between digital health literacy and knowledge (p = 0.000) and behavior (p = 0.000) regarding hand hygiene.

**Conclusion:**

there is an association between higher digital health literacy scores and hand hygiene knowledge and behavior.

## Highlights

**(1)** Average digital health literacy of Brazilian adolescents was 29.89 points

**(2)** Average knowledge on hand hygiene was 13.1 points and behavior was 11.1.

**(3)** Knowledge and behavior scores were lower among men.

**(4)** Higher digital health literacy in adolescents who did not wear masks when playing sports.

**(5)** There is an association between higher digital health literacy scores and hand hygiene knowledge and behavior.

## Introduction

During the COVID-19 (coronavirus disease) pandemic, there was an avalanche of information that generated uncertainty and limitations in decision-making by the population; for protection strategies to be successful, information related to COVID-19 needed to be understood and followed^1^ ^)-(^ ^2^.

The practice of hand hygiene was one of the main strategies recommended for prevention during the COVID-19 pandemic^3^. National campaigns on the subject were promoted^3^, recommending hand hygiene with soap and water^4^ or using alcohol in its 70% formulation, which is recommended when hands do not have dirt visible to the naked eye^4^.

Each phase of the pandemic presented different challenges, also differing between countries^5^. In Brazil, five phases were distinguished from March 2020 to March 2022^5^. In the first (from March to August 2020), preventive hand hygiene strategies, use of alcohol gel and face masks in public places, as well as social distancing, were implemented. The second phase (from September 2020 to January 2021), in addition to relying on preventive strategies, was marked by the beginning of a second “wave” of the pandemic, in January 2021. In the third phase (from February to June 2021), there was a widespread crisis in the health system, the circulation of different variants of the virus and gradual vaccination. In the fourth phase (from July to November 2021), there was a better balance in the health system with effective vaccination and a reduction in virus transmission. In the fifth phase (from December 2021 to March 2022), a new variant (*omicron*) appeared, and vaccination continued^5^. Finally, on May 5^th^, 2023, the World Health Organization (WHO) declared the end of the COVID-19 pandemic. Currently, in Brazil, the scenario is one of circulation of different variants of the virus, coverage of a large part of the population by at least two doses of vaccines and evidence of adequate treatment^6^.

The infodemic related to COVID-19 was a challenge present in all phases of the COVID-19 pandemic and persists to this day^7^. The infodemic was characterized by an excess of information, including incorrect or outdated information, shared digitally, from person to person and through other means and communication channels^8^.

During the COVID-19 pandemic, the World Health Organization highlighted the importance of actions to promote digital health literacy (DHL) in specific and vulnerable populations^8^. The search for DHL, which provides reliable information and its appropriate use, applies especially to adolescents who are constant users of the Internet and social networks^1^. Healthy behavior in this period of life depends more on individual decisions and life circumstances than on guidance and help from parents or adults^9^ ^)-(^ ^10^; furthermore, learning and habits acquired during adolescence can have repercussions throughout adulthood.

Norman and Skinner define DHL as “the ability to search, find, understand and evaluate health information from electronic sources and apply the acquired knowledge to address or solve a health problem” and proposed, to measure this ability, the eHealth scale Literacy Scale (eHEALS)^11^. In Brazil, it was cross-culturally adapted^12^ ^)-(^ ^13^ showing good psychometric properties.

During the pandemic period, in a study in Norway with 2,205 adolescents aged 16 to 19 years old, it was found that hand hygiene, physical distancing and limiting the number of social contacts were the measures most reported by adolescents; In addition, the association between health literacy (HL) and knowledge and behavior related to hand hygiene was highlighted, as well as the association between greater HL and less socialization^2^. A qualitative study conducted by the same researchers reveals that adolescents understood and adopted protective measures, such as hand hygiene and social distancing, that they knew how to find reliable information, that they were attentive to it and did their best to follow the recommendations for preventing COVID-19^14^.

Despite the great importance of DHL as an essential competence to deal with health threats, such as the COVID-19 pandemic^2^ ^),(^ ^15^ ^)-(^ ^16^, there is limited evidence on the topic among Brazilian adolescents^17^ ^)-(^ ^19^. Thus, the present study aimed to investigate factors associated with digital health literacy, knowledge and behavior for hand hygiene among Brazilian adolescents during the COVID-19 pandemic.

## Method

### Study design and period

This is a cross-sectional and analytical study, carried out with Brazilian adolescents from the five macro-regions of the country, guided by the Strengthening the Reporting of Observational Studies in Epidemiology (STROBE)^20^ guideline and carried out from June 30^th^ to August 8^th^, 2021 (a period in which Brazil was in transition between the third phase and fourth phase of the pandemic), virtually, using the Google Forms tool. 

### Study population and sample

The target population was made up of Brazilian teenagers between 15 and 19 years old, with access to the internet. Those whose forms failed to be completed were excluded. It is noteworthy that the eligible age group was determined due to the fact that the Ministry of Health follows the definition of adolescence established by the World Health Organization (WHO): period between 10 and 19 years old^21^. Furthermore, we consider the fact that eHEALS in the Brazilian version has been validated in individuals aged 15 and over^12^. For the sample calculation, a non-probabilistic intentional sample was used due to the absence of a referenced population and the respondent’s interest in participating in the research. To calculate the sample estimate, simple random sampling was considered, type I and II errors equal to a maximum of 0.05 and 0.20, respectively, linear correlation between eHEALS scores and hand hygiene knowledge score estimated at 0.15 and the presence of five confounding variables in the statistical analysis models. With these assumptions, at least 387 participants were estimated. The sample consisted of 483 adolescents, five of whom refused to participate in the research and five were excluded due to errors in filling out the questionnaires, resulting in a final sample of 473 participants.

### Study variables

#### Outcomes

Assessment of knowledge and behavior regarding hand hygiene according to the Brazilian version of the Questionnaire on Handwashing Knowledge and Behavior (QHKB-BR) and digital health literacy by eHEALS.

#### Independent variables

Sociodemographic variables: gender (male and female), region of residence (North, Northeast, Central-West, Southeast and South), age (in years), education (non-literate, primary education I or II, secondary education, undergraduate degree), be studying or have studied a technical course or degree in the health area and mention that one of those responsible is a health professional.

### Data collection instruments

A virtual data collection instrument was developed on the Google Forms platform by the authors (https://www.google.com/intl/en-GB/forms/about/), containing variables related to adolescents regarding COVID-19: COVID-19 diagnosis (yes or no); death of a family member due to COVID-19 (yes or no); situation in which you do not wear masks (meetings with friends and family, playing sports, nightclubs, parties or bars, home, bars, outdoors and a space to present other places); whether they were looking for information about the pandemic (yes or no); place to search for information about the pandemic (Television (TV), radio, newspapers, podcasts, schools, YouTube, Twitter (X), Snapchat, TikTok, Instagram^®^, Facebook^®^, other media, family, friends, scientific articles, university , does not seek information) and the desire to vaccinate (no or yes; yes, had already received one dose of the vaccine; had already received two doses of the vaccine or a single dose of the vaccine).

As open questions, adolescents had to answer their reason for not wanting to be vaccinated (when applicable), two COVID-19 prevention measures they adopted and two measures they had difficulty carrying out; two pieces of information they had about the COVID-19 vaccine and two pieces of information they would like to obtain.

In Part 3 of the instrument, the answers were related to the QHKB-BR^22^ and eHEALS^11^.

The Questionnaire on Handwashing Knowledge and Behavior (QHKB) was developed in Norway to assess hand hygiene in adolescents, containing six items, divided into two domains: knowledge (items 01 to 03) and behavior (items 04 to 06). Each item is measured on a five-level ordinal scale (“totally disagree”, “disagree”, “neither disagree nor agree”, “agree” and “totally agree”)^2^. The QHKB presented a high Cronbach’s alpha (0.75 for knowledge and 0.76 for behavior)^2^. The QHKB-BR was adapted to the Brazilian context and the reliability analysis demonstrated an alpha (α) equal to 0.81 and an omega (ωt) of 0.81. The QHKB-BR showed high internal consistency for a one-dimensional instrument, unlike the original version, which was high for a two-dimensional instrument^22^. The assessment of knowledge and behavior in relation to hand hygiene occurred using the QHKB-BR, with both the knowledge and behavior domain scores totaling 3 to 15 points each^22^.

eHEALS was one of the first scales developed in Canada to assess consumers’ perceived skills in using information technology, in 664 young Canadians aged 13 to 21^11^. It consists of eight items, aimed at measuring individuals’ perceived knowledge, comfort and skills in finding, evaluating and applying electronic health information to health problems^11^. The response options are arranged on a five-item Likert scale, ranging from 1 (totally disagree) to 5 (totally agree), with a total of 8 to 40 points^11^. Higher scores on eHEALS presuppose that the individual has higher levels of DHL^11^. In Brazil, when applied to individuals aged 15 to 55 years, it presented Cronbach’s α = 0.89^12^. In factor analysis, an adequate sample size was observed (KMO = 0.89) with a non-identity correlation matrix (Bartlett’s test of sphericity, p < 0.001) and without the influence of multicollinearity (determinant = 0.026). The unidimensionality of eHEALS was confirmed by extracting just one factor (eigenvalue = 4.43), representing 55.36% of the instrument’s total variance^12^. In individuals aged between 18 years and 80 years, the eHealth Literacy Scale - Brazilian version (eHEALS-Br) showed excellent internal consistency (α = 0.95 and ω = 0.95), only one dimension and explained variance of 81,79^13^.

### Data collection

Participants were recruited through the dissemination of the digital form distributed by five researchers (north and southeast regions) and two collaborators (north and south regions), on social media and personal contacts (WhatsApp and e-mail). Invitations were sent out, which were multiplied using the “snowball” technique^23^. The researchers disseminated the research at universities, churches, municipal and state health departments. A brief description of the study and its objectives was provided to those responsible for adolescents under 18 years of age, so that adults could pass on the information to young people. Adolescents aged 18 or over were invited directly by the authors. Data collection was interrupted after reaching the sample size calculation with the participation of adolescents from 26 states and the Federal District.

### Data analysis

Multiple linear regression models with normal response were adjusted to explain the scores of the QHKB-BR and eHEALS instruments as a function of the independent variables that were statistically associated with p<0.20 in the bivariate analysis. In the final model, associations were considered statistically significant if p<0.05. Analyzes were carried out using SPSS 21 software. For open-ended questions, the contents were read by two researchers, supervised by a third researcher, and analyzed whether they corresponded to WHO recommendations^24^. Subsequently, they were grouped by similarities and transported to an Excel spreadsheet. In the end, each group of responses was read exhaustively to check whether they were indeed related to each other, a theme was identified that could characterize the group of responses and a descriptive analysis was carried out. 

### Ethical aspects

The research was approved by the Research Ethics Committee of the responsible institution, under CAAE number 45521121.1.0000.5411 and opinion number 4.661.979. Acceptance to participate in the research was registered through the Free and Informed Consent Form in digital format in the case of adolescents over 18 years of age or parents/guardians of children under 18 years old. Participants under 18 years of age, in addition to their parents’ permission, were asked to accept the Free and Informed Assent Form, also in digital format. 

## Results

Regarding the DHL of Brazilian adolescents, the average score was 29.89 points (SD±5.30) and a median of 30.0 (minimum of 12 and maximum of 40). For knowledge about hand hygiene, it was 13.1 points (SD±1.5) and a median of 13.0 (minimum of 7 and maximum of 15). For behavior, the average was 11.1 points (SD±2.7) and median was 11.0 (minimum of 3 and maximum of 15). For the total score of hand hygiene knowledge and behavior, the average was 24.3 points (SD±3.6), with a median of 24.0 (minimum of 14 and maximum of 30). The characterization and information of adolescents regarding COVID-19 are presented in Table 1.

**Table 1:** Sociodemographic characterization of Brazilian adolescents and information regarding COVID-19 (n = 473). Brazil, 2023

Variables	Total	
	n*	%^†^
**Gender**		
Female	314	66,4
Male	155	32,8
Other	4	0,8
**Region**		
Southeast	369	78,0
North East	46	9,7
North	26	5,5
Midwest	18	3,8
South	14	3,0
**Age (in years)**		
18-19	267	56,4
15-17	206	43,6
**Education**		
High school in progress	214	45,2
College in progress	146	30,9
Finished high school	55	11,6
Preparatory course	36	7,6
Secundary education in progress	22	4,7
Bachelor’s degree or technical course in the healthcare field	88	18,6
Paid activity	157	33,2
Parents or guardians are in the healthcare field	114	24,1
**Education of parents/guardians**		
High school	176	37,2
Graduation	121	25,6
Postgraduate	110	23,3
Secundary school	40	8,5
Elementary school	23	4,9
Not literate	3	0,6
Diagnosis of COVID-19	81	17,1
Death of a family member due to COVID-19	21	4,4
**Situation in which a mask was not wore**		
Home	431	91,7
Family reunions	264	55,8
Outdoor environment	152	32,3
Practicing sport	94	19,9
Parties	23	4,9
Work	11	2,3
Others	17	3,6
Search for information about the pandemic	379	80,1
**Places where they search for information**		
Social media^‡^	443	93,6
Television	272	57,4
Newspapers	157	33,1
Family	149	31.4
Scientific articles	115	24,3
Other media^§^	97	20,5
Friends	95	20,5
School	95	20,0
Faculty	68	14,4
Radio	25	5,28
Websites	11	2,32
**Desire to receive vaccine**	444	93,9

^*^N = Total number of participants; ^†^% = Percentage; ^‡^Instagram^®^, Facebook^®^, Twitter (X), TikTok, Snapchat, podcasts, YouTube; ^§^Other media classified by teenagers that were not part of the questionnaire selection board

Regarding the preventive measures adopted by the participants, it is noteworthy that 4.4% (n = 21) did not respond to the question asked, and the others responded with one or more preventive measures, totaling 895 prevention measures cited. Of these, all are in accordance with the prevention measures adopted by the WHO^24^, except the use of disposable gloves and the use of two masks (Table 2).

**Table 2:** Distribution of adolescents’ responses regarding the preventive measures used and not used to prevent COVID-19 and the information they know or would like to know about vaccines for COVID-19. Brazil, 2023

Variable	n*	%^†^
**Preventive measures adopted (N=895)**		
Face mask	379	83,8
Hand hygiene with 70% alcohol	252	55,7
Hand hygiene with soap and water	91	20,1
Social distancing	75	16,6
Social isolation	67	14,8
Personal or environmental hygiene	28	6,2
Others^‡^	03	0,7
**Difficulties in adopting preventive measures (N=406)**		
Social distancing	83	21,7
Face mask	73	19,1
No difficulties	62	16,2
Social isolation	66	17,2
Use of alcohol or alcohol gel	46	12,0
Personal or environmental hygiene	56	14,7
Sanitization of hands	18	4,7
Others^§^	02	0,5
**Information they know about vaccines (N=402)**		
Vaccine effectiveness	102	24,11
Reduces serious conditions	66	15,60
Side effect	45	10,63
Vaccine production	42	9,92
Benefits of vaccines	28	6,61
Others^||^	25	5,91
Mass vaccination	24	5,67
Vaccine doses	23	5,43
Negative information	19	4,49
They do not know	16	3,78
Vaccine needed to fight the pandemic	12	2,83
**Information you would like to know (N=319)**		
Vaccine effectiveness	98	29,96
Different types of vaccines	60	18,34
Vaccination in teenagers	50	15,29
None	33	10,09
Side effect	29	8,86
Post-vaccine	18	5,50
Required doses	13	3,97
Others^¶^	10	3,05
Security and distribution	08	2,44

*N = Frequency; ^†^% = Percentage; ^‡^Have contact with people who follow preventive measures, go to the doctor in case of symptoms, use gloves); ^§^Nasal swab and use of gloves; ^||^Second dose is a booster; it takes 15 days to take effect; are following the vaccination schedule, among others; ^¶^Advancement of vaccines, reasons for not vaccinating children under 12 years of age, post-vaccine cases, among others

Regarding difficulties in applying preventive measures, 19.9% (n = 96) did not respond to this question, and the rest responded with one or more answers, totaling 406 difficulties in adopting preventive measures. Social distancing was the preventive measure that adolescents highlighted as the greatest difficulty (21.7%; n = 83) (Table 2).

Regarding information from adolescents about vaccines to prevent COVID-19, 402 responses were obtained, grouped into 11 main themes, by one of the researchers using an Excel spreadsheet; each category was named according to the answers cited by each adolescent, with emphasis on information about the effectiveness of the vaccine (102; 24.11%) and reduction in the severity of COVID-19 (66; 15.60%). Regarding the information that teenagers would like to obtain, 319 answered this question, totaling seven themes. From the analysis of the responses, it was observed that the effectiveness of the vaccine was the topic of greatest interest to adolescents (29.96%; n = 98) (Table 2).

Table 3 presents the bivariate association and multiple linear regression adjusted to explain the QHKB-BR outcome. The multiple analysis shows that QHKB-BR was higher: among adolescents who had completed high school (b = 2.28 CI95%=(1.13; 3.43); p=0.000) compared to those with college, among adolescents who attended degree in the health area (b = 1.16 IC95%=(0.19; 2.13); p=0.019), among those seeking information about the pandemic (b = 0.97 IC95%=(0.16; 1.78); p=0.019) and among those seeking information via scientific articles (b = 1.23 95%CI=(0.38; 2.08); p=0.005). And, in addition, the QHKB-BR score was lower: among men (b = -0.70 95%CI= (-1.37; -0.02); p=0.042) and among adolescents who reported not wearing a mask when open air (b = -0.90 CI95%= (-1.57; -0.23); p=0.009).

**Table 3:** Adjusted linear regression to explain the QHKB-BR* outcome (n = 473). Brazil, 2023

Variables	Bivariate associations				Multiple regression			
	b^†^	CI^‡^95%		*p* ^§^	b^†^	CI^‡^95%		*p* ^§^
**Gender**								
Others^||^	-0,80	-4,35	2,74	0,657	-0,72	-4,68	3,25	0,723
Male	-0,83	-1,52	-0,14	0,019	-0,70	-1,37	-0,02	0,042
Female^¶^								
**Region**								
Other regions	-0,43	-1,21	0,36	0,288				
Southeast**								
**Age**								
18-19 years old	0,09	-0,57	0,75	0,786				
15 to 17 years^††^								
**Education**								
Preparatory course	-0,34	-1,64	0,96	0,609	0,53	-0,81	1,88	0,438
Elementary school II completed	-0,12	-1,72	1,49	0,887	1,21	-0,43	2,86	0,148
High school in progress	0,27	-0,48	1,02	0,485	1,13	0,26	2,00	0,011
Finished high school	1,77	0,66	2,87	0,002	2,28	1,13	3,43	0,000
College in progress^‡‡^								
Undergraduate courses in the health sector	0,71	-0,13	1,55	0,096	1,16	0,19	2,13	0,019
**Work**								
Yes, outside the house	-0,43	-1,16	0,30	0,251				
Yes, at home office	0,34	-1,13	1,81	0,647				
Work^§§^								
Parents/caregivers	-0,49	-1,25	0,27	0,208				
**Education of parents or guardians**								
Postgraduate	2,28	-1,83	6,39	0,277				
Graduation	1,09	-3,02	5,19	0,604				
High school	1,84	-2,25	5,92	0,378				
Elementary school II	0,98	-3,22	5,18	0,646				
Elementary school I	0,68	-3,63	4,99	0,757				
Not educated^||||^								
**Diagnosis of COVID-19**	-0,51	-1,38	0,35	0,247				
**Family death due to COVID-19**	-0,64	-2,22	0,95	0,431				
**Situations in which do not use masks**								
Family reunions	-0,25	-0,91	0,41	0,462				
Practicing sport	0,05	-0,77	0,87	0,914				
Parties	-0,25	-1,77	1,27	0,745				
Home	0,73	-0,46	1,92	0,229				
Work	-1,31	-3,48	0,85	0,235				
Outdoor environment	-0,93	-1,63	-0,24	0,008	-0,90	-1,57	-0,23	0,009
Others^¶¶^	1,60	-0,15	3,35	0,074	1,83	0,07	3,60	0,042
**Search for information about the pandemic**	1,40	0,58	2,21	0,001	0,97	0,16	1,78	0,019
Places where they look for information								
Scientific articles	1,49	0,74	2,24	0,000	1,23	0,38	2,08	0,005
Universities	1,10	0,18	2,03	0,020	0,06	-1,01	1,13	0,913
**Desire to receive vaccine**	0,96	-0,45	2,37	0,181	0,43	-0,95	1,81	0,541

*Brazilian version of the Questionnaire on Handwashing Knowledge and Behavior; ^†^Intercept; ^‡^Confidence interval; ^§^Bivariate analysis by simple/multiple linear regression; ^||^Gender classification of sex, other than male or female; ^¶^Reference to sex; **Reference to regions in Brazil; ^††^Reference for age; ^‡‡^Reference for education levels; ^§§^Reference for work; ^||||^Reference to the education levels of parents or guardians; ^¶¶^Classified by adolescents who were not part of the questionnaire selection panel (vaccinated close family members, car, during meals, being alone, small groups of people)

Table 4 presents the bivariate association and multiple linear regression adjusted to explain the eHEALS outcome. In the multiple analysis, the association of higher DHL scores in adolescents who did not wear a mask while playing sports was evident (b = 1.25 95%CI= (0.23; 2.27); p=0.017). There was an association between DHL and knowledge (p =0.000) and behavior (p =0.000) regarding hand hygiene. It was found that the greater the knowledge and behavior regarding hand hygiene, the higher the eHEALS score, generating an increase in the score of 1.28 and 0.40, respectively.

**Table 4:** Adjusted linear regression to explain the eHEALS* outcome (n = 473). Brazil, 2023

Variable	Bivariate associations				Regressão múltipla			
	b^†^	CI^‡^ 95%		p^§^	b^†^	CI^‡^ 95%		p^§^
**Gender**								
Male	-2,90	-8,12	2,32	0,276				
Female	0,05	-0,97	1,07	0,928				
Others^||^								
**Region**								
Midwest	3,37	0,20	6,53	0,037				
North East	0,97	-1,57	3,50	0,454				
South	2,28	-1,14	5,70	0,191				
Southeast	1,96	-0,14	4,05	0,067				
North^¶^								
Other regions	-0,638	-1,790	0,514	0,277	-0,24	-1,23	0,76	0,639
**Age**								
18-19 years old	1,28	0,32	2,24	0,009	0,96	-0,62	2,54	0,235
15 to 17 years**								
Age (in years)	0,42	0,09	0,75	0,013				
**Education**								
Preparatory course	-1,14	-3,04	0,76	0,241	-0,83	-2,58	0,92	0,352
Elementary II education in progress	-2,03	-4,36	0,31	0,089	-0,43	-3,04	2,19	0,749
High school in progress	-0,73	-1,83	0,36	0,189	0,20	-1,54	1,95	0,820
Finished high school	2,07	0,46	3,69	0,012	0,89	-0,63	2,40	0,251
Faculty^††^								
Courses in the health sector	1,14	-0,09	2,36	0,068	0,42	-0,85	1,69	0,514
**Work**								
No	0,08	-1,00	1,15	0,891				
Yes, outside the home	0,85	-1,30	3,01	0,438				
Yes, at home office^§§^								
Parents/caregivers	-0,20	-1,32	0,91	0,719				
**Education parents or guardians**								
Postgraduate	3,62	-2,40	9,64	0,238				
Graduation	2,39	-3,62	8,41	0,435				
High school	1,93	-4,06	7,92	0,527				
Elementary school II	2,37	-3,79	8,53	0,451				
Elementary school I	3,80	-2,52	10,11	0,239				
Not literate^||||^								
**Diagnosis of COVID-19**	-0,24	-1,51	1,03	0,708				
**Death of a family member due to COVID-19**	-0,09	-2,42	2,23	0,936				
**Situations in which did not use masks**								
Family reunions	0,06	-0,91	1,03	0,902				
Playing sports	1,24	0,04	2,43	0,042	1,25	0,23	2,27	0,017
Parties	1,01	-1,22	3,23	0,375				
Houses	-0,35	-2,09	1,39	0,696				
Work	0,83	-2,34	4,01	0,606				
Outdoor environments	-0,36	-1,39	0,66	0,485				
Others^¶¶^	0,09	-2,48	2,66	0,945				
**Search for information about the pandemic**	1,63	0,43	2,83	0,008	0,53	-0,53	1,59	0,323
**Places where they search for information**								
Scientific articles	1,91	0,81	3,02	0,001	0,21	-0,90	1,32	0,707
Universities	1,93	0,57	3,28	0,005	0,66	-0,73	2,06	0,352
Desire to receive vaccine	-1,17	-3,23	0,89	0,267				
QHKB-BR*** Knowledge	1,69	1,41	1,97	0,000	1,28	0,97	1,59	0,000
QHKB-BR*** behavior	0,78	0,62	0,95	0,000	0,40	0,22	0,57	0,000

*eHealth Literacy Scale; ^†^Intercept; ^‡^Confidence interval; ^§^Bivariate analysis by simple/multiple linear regression; ^||^Reference to sex; ^¶^Reference for regions in Brazil; **Reference for age; ^††^Reference for education levels; ^§§^Reference for work; ^||||^Reference to the education levels of parents or guardians; ^¶¶^Classified by adolescents who were not part of the questionnaire selection panel (vaccinated close family members, car, during meals, being alone, small groups of people); ***Brazilian version of the Questionnaire on Handwashing Knowledge and Behavior

## Discussion

The highlight of the present study was to seek evidence, through validated instruments and open questions, that could contribute to the DHL assessment of Brazilian adolescents at such a challenging time for all populations and provide bases for directing public policy actions in the country. Researchers consider that LS can have a positive impact on controlling the global spread of COVID-19, however, it is necessary for individuals to be trained in acquiring information in addition to understanding and knowing how to apply the knowledge acquired^25^.

In the present study, the majority of adolescents were female, however this variable was not associated with DHL. Our findings are in line with those found in the study conducted in Brazil, in which the gender variable was not associated with LS^26^. On the other hand, there was an association between lower QHKB-BR scores in men, compared to women. Scholars believe that women are more likely to perceive COVID-19 as a health problem and end up adhering more to restrictive public policy measures; and these authors also think that gender differences in attitudes and behaviors are considerable in all countries^27^.

The majority of adolescents in this study cited the face mask as a preventive strategy adopted during COVID-19, however, when questioned in an open question they reported having difficulties using it. In response to preventing COVID-19, the WHO already included non-pharmaceutical interventions, such as wearing a mask, hand hygiene and social distancing, to reduce viral transmission^28^. Research carried out in China^29^ argues that, although the use of a mask and hand hygiene are strategies used to prevent COVID-19, educational background, mother’s education and place of residence are associated with better use behavior wearing a mask and hand hygiene^29^. In another investigation carried out in Turkey^30^ with participants over 18 years of age, it was found that hand hygiene habits vary by age and gender, while social distancing and mask use scores vary only by gender^30^.

Although the QHKB-BR scores were satisfactory for knowledge and behavior regarding hand hygiene, when the adolescents were questioned in an open-ended question, they mentioned cleaning with alcohol gel more frequently than cleaning with soap and water. One of the assumptions would be that teenagers understand that using alcohol gel would be more important than washing their hands with soap and water. These data indicate the importance of educational actions that promote HL in adolescents, including guidelines for hand hygiene, which clarify when handwashing with soap and water is recommended and when this can be replaced by the use of alcohol gel. A review of the literature highlighted the importance of health professionals playing a key role in good communication during educational interventions and health education actions with fathers/mothers/caregivers, aiming to promote child development^31^.

Although hand hygiene is a protective, inexpensive and widely available recommendation for personal protection and epidemic prevention, our findings confirm that there are still challenges in adherence. Thus, interesting strategies can be found in the international literature that corroborate the theme. A study carried out in China^32^ and India^32^ on the costs and benefits of handwashing behavior change programs suggests large economic gains related to reductions in epidemic outbreaks through behavior change programs in homes^32^. Research carried out in Turkey^33^, including high school students, evaluated the behavior of hand hygiene and mask use, showing the importance of increasing, through training, the awareness of parents and school-age children about preventive measures, such as correct hand hygiene technique (time, frequency), selection of appropriate mask, as well as how to use and dispose of it^33^.

Regarding the information that adolescents would like to have about vaccines to prevent COVID-19, it was observed that the effectiveness of the vaccine was the topic of greatest interest among participants. This led to the interest of most teenagers in receiving the vaccine, a fact that we do not know if this occurred. However, caution must be taken when analyzing this information, and it is necessary to highlight that vaccination for this group was still in its infancy, that a large part of the participants were from the southeast region, and that 24.1% (n=114) of those responsible were health professionals. It is also noteworthy that there was no association between DHL and QHKB-BR and the desire to receive the vaccine. In line with our findings, low vaccine hesitancy was identified in a study with 526 Brazilian adolescents aged 14 to 19 years. In the study, LS also did not influence the intention not to be vaccinated, but rather the perception of the threat of COVID-19^19^.

In this investigation, it was found that the majority of teenagers sought information about the pandemic on social media, scientific articles and at college. However, there was no association between DHL and the place where adolescents sought information. On the other hand, there was an association between higher QHKB-BR scores and the search for information about the pandemic and the search for information in scientific articles. In a study carried out in Australia with adolescents aged 12 to 17 years old, it was identified that online resources accessed to unintentionally search for health information on social media did not prove to be effective in DHL for adolescents, but rather the use of heuristic strategies taught at school for general internet research^26^. However, the authors state that some strategies are inappropriate for the context of digital health information, as the terminology makes it difficult to understand or evaluate online health information^26^. A survey carried out in Pakistan showed that teenagers are inclined to use digital platforms to search for health information^34^.

Our findings correspond to recent findings showing that adolescents with higher DHL measured by eHEALS have higher knowledge (p = 0.000) and behavior scores regarding hand hygiene (p = 0.000). In line with the findings of the present study, are the results of a recent survey carried out in Pakistan^34^ including 387 adolescents, 57.1% female, 15.8 years old (±1.50), whose objective was to evaluate the association between health protective behavior and LS to prevent the spread of COVID-19. The authors also found an association between HL and health knowledge (p=0.023); and between HL and health behavior (p < 0.001)^34^. Work conducted in Poland^35^ corroborates our study, showing that hand hygiene is associated with an influence on adolescents’ knowledge and behavior during the course of the COVID-19 pandemic^35^. A study carried out in Norway^2^ points out that there was an association between LS and knowledge and behavior of washing hands measured by the QHKB.

A cross-sectional study including 1,322 Japanese university students showed that high levels of HL are associated with healthy lifestyle habits and, the greater the students’ participation in classes related to health education, the better their competence in accessing and understanding health information in the health promotion domain. health^36^. Therefore, it is considered relevant to promote continuous strategies in schools, not just isolated actions, to improve HL among adolescents.

Interestingly, this research showed an association between higher DHL scores and not using a mask while practicing sports. In this sense, even knowing that wearing a mask was mandatory, a possible explanation would be the fact that the DHL seems to have contributed to the adolescents’ decision-making. A review of the literature indicated that HL can interfere with adolescents’ decision-making and that a low HL can lead to decisions and actions that expose this population physically and mentally, in addition to consequences that can compromise their personal, professional and family future^18^. 

The results of this study provide evidence for understanding the importance of DHL, which influences knowledge and, consequently, hand hygiene behavior. To improve adolescents’ HL, it is important to develop educational strategies in schools, aimed at facing the threat of sudden epidemics of serious infectious diseases. The HL of adolescents has six dimensions related to this stage of development (i.e., six “Ds”), which need to be considered in the planning and execution of health actions: demographic patterns (adolescents are particularly vulnerable to social and health inequalities) , differential epidemiology (some diseases and health risks are highly age-specific for adolescents), developmental change (e.g., biological, cognitive, psychological), dependence (on parents and peer groups), democratic citizenship (adolescents are social actors in their own right) and digitalization (digital media is an integral component of their daily lives)^37^. A study carried out in Germany with students between 16 and 20 years old, to evaluate a course on an e-learning platform, showed that, after completing the course, teenagers achieved higher levels of HL, suggesting that DHL should be part of the school curriculum as part of the mandatory structure for digital education, with the potential to promote HL in school-age children^38^, a fact that could positively impact their health in adolescence and adulthood. Likewise, an editorial published in Lancet, authored by experts in the field of HL, advocates taking advantage of the potential of schools to educate children about health and improve school processes and structures, aiming at both behavioral and social change^39^.

Authors report that social and environmental conditions play an important role in public health^40^. They also state that HL is considered directly or indirectly linked to health outcomes, as it is one of the Social Determinants of Health^41^. Furthermore, low HL can lead the population to be less likely to use preventive measures and services aimed at communicable and non-communicable diseases, in addition to being less receptive to health education messages^42^ ^)-(^ ^43^.

Some limitations of the present research need to be stated. Data collection was completed in August 2021, when vaccination began among adolescents, which may have guided responses about vaccination. This collection was carried out virtually, and adolescents without access to electronic resources (computers, telephones and Wi-Fi) and those without reading skills were unable to participate. Also, we did not investigate the socioeconomic level of guardians and adolescents to associate it with LS in the levels of prevention and control of the COVID-19 pandemic.

The findings of this research contribute to adolescent health in Brazil, providing bases for comparison with other countries. It is recommended that health professionals should work in schools and universities in activities aimed at developing HL. Furthermore, they must be involved in the development of appropriate and quality educational resources, contributing to adolescents’ DHL. Finally, it is still necessary to provide educational activities on the practice of hand hygiene with soap and water and alcohol gel aimed at adolescents. 

## Conclusion

The desire to receive the COVID-19 vaccine was the most reported preventive measure, followed by the use of face masks, cleaning with alcohol gel; and hand hygiene with soap and water is much less mentioned. The effectiveness of the vaccine was the topic that aroused the most interest and about which teenagers said they had the most information.

There is an association between DHL and hand hygiene knowledge and behavior. The DHL influenced adolescents’ knowledge and behavior regarding hand hygiene and not using a mask during sports. Hand hygiene knowledge and behavior were higher among adolescents who had completed high school compared to those attending higher education, among those attending a health course, among those seeking information about the pandemic and among those seeking information via articles scientific.

The study suggests that educational strategies related to the importance of hand hygiene and aimed at adolescents are still important. We believe that educational actions must be continuous and started from the first years of life, with intersectoral actions that bring together health and education professionals.
