# Supplementary material for: Digital health literacy, behavior and knowledge of adolescents for hand hygiene during the COVID-19 pandemic
Source: Rev Lat Am Enfermagem. 2024 Aug 30;32:e4308. doi: 10.1590/1518-8345.7228.4308 (PMC11368072; doi:10.1590/1518-8345.7228.4308)
Supplement: Translation

# Translation: Letramento digital em saúde, comportamento e conhecimento de adolescentes para higienização das mãos na pandemia de COVID-19*

**DOI:** 10.1590/1518-8345.7228.4309

**Authors:** Rafaela Aparecida Prata, Juliana Bastoni da Silva, Sidiany Mendes Pimentel, Hélio Rubens de Carvalho Nunes, Marla Andréia Garcia de Avila

**Affiliations:** 1 Universidade Estadual Paulista, Departamento de Enfermagem, Botucatu, SP, Brasil.; 2 Universidade Federal do Tocantins, Departamento de Enfermagem, Palmas, TO, Brasil.

**Keywords:** Letramento em Saúde, Desinfecção das Mãos, Higiene das Mãos, COVID-19, Adolescente, Saúde do Adolescente

## Abstract

**Objetivo:**

investigar fatores associados ao letramento digital em saúde, o conhecimento e comportamento para higienização das mãos de adolescentes brasileiros na pandemia de COVID-19.

**Método:**

estudo transversal realizado com 473 adolescentes de 15 a 19 anos. Os dados foram obtidos virtualmente, entre junho e agosto de 2021, por meio de questionário sobre características sociodemográficas, letramento digital em saúde, conhecimento e comportamento para higienização das mãos. Variáveis foram ajustadas por modelos de regressão linear múltipla com resposta normal.

**Resultados:**

a pontuação média de letramento digital em saúde foi de 29,89 ±5,30. Para o conhecimento e comportamento na higienização das mãos foi de 13,1 ±1,5 e 11,1±2,7, respectivamente. Maiores pontuações de conhecimento e comportamento na higienização das mãos se associaram ao ensino médio finalizado, isto se comparados aos que cursam faculdade, aos que frequentam curso em saúde, aos que buscam informações sobre a pandemia e em artigos científicos. Além disso, houve maiores pontuações do letramento digital em saúde em adolescentes que não utilizavam máscaras quando praticavam esporte (p= 0,017). Houve associação entre letramento digital em saúde e conhecimento (p = 0,000) e comportamento (p = 0,000) para higienização das mãos.

**Conclusão:**

existe associação entre maiores escores de letramento digital em saúde e o conhecimento e comportamento para a higienização das mãos.

## Destaques

**(1)** Média do letramento digital em saúde de adolescentes brasileiros foi de 29,89 pontos.

**(2)** Média de conhecimento na higienização das mãos foi de 13,1 pontos e de comportamento, 11,1.

**(3)** Pontuação de conhecimento e comportamento foi menor entre os homens.

**(4)** Maior letramento digital em saúde nos adolescentes que não utilizavam máscaras quando praticavam esporte.

**(5)** Associação entre letramento digital em saúde e conhecimento e comportamento para a higienização das mãos.

## Introdução

Durante a pandemia de COVID-19 (*coronavirus disease*), houve uma avalanche de informações que geraram incertezas e dificuldades na tomada de decisões pela população. Dessa forma, para que as estratégias de proteção fossem bem-sucedidas, as informações relacionadas à COVID-19 precisavam ser compreendidas e cumpridas^1^ ^)-(^ ^2^. 

A prática de higienização das mãos foi uma das principais estratégias recomendadas para a prevenção na pandemia da COVID-19^3^. Campanhas nacionais sobre a temática foram promovidas^3^, recomendando a higienização das mãos com água e sabão^4^ ou a utilização do álcool em sua formulação 70%, indicado quando as mãos não apresentam sujidades visíveis a olho nu^4^.

Cada fase da pandemia apresentou diferentes desafios, diferindo também entre os países^5^. No Brasil, distinguiu-se cinco fases de março de 2020 a março de 2022^5^. Na primeira (de março a agosto de 2020) implementaram-se estratégias preventivas de higiene das mãos, uso de álcool em gel e de máscaras faciais em locais públicos, bem como distanciamento social. A segunda fase (de setembro de 2020 a janeiro de 2021) além de contar com as estratégias preventivas, foi marcada pelo início de uma segunda “onda” da pandemia, em janeiro de 2021. Na terceira fase (de fevereiro a junho de 2021) sucedeu uma crise generalizada do sistema de saúde, circulação de diferentes variantes do vírus e vacinação gradativa. Na quarta fase (de julho a novembro de 2021) ocorreu um melhor equilíbrio do sistema de saúde com efetividade da vacinação e redução da transmissão do vírus. Na quinta fase (de dezembro de 2021 a março de 2022) apareceu uma nova variante (ômicron), e continuou-se a vacinação^5^. Por fim, em 5 de maio de 2023, a Organização Mundial da Saúde (OMS) decretou o fim da pandemia COVID-19. Atualmente, no Brasil, o cenário é de circulação de diferentes variantes do vírus, cobertura de grande parte da população por pelo menos duas doses de vacinas e evidências de um tratamento adequado^6^.

A infodemia relacionada à COVID-19 foi um desafio presente em todas as fases da pandemia COVID-19 e persiste até os dias atuais^7^. Caracterizada pelo excesso de informações, a infodemia consistiu na disseminação de informações incorretas ou desatualizadas, compartilhadas digitalmente, de pessoa a pessoa e por outros meios e canais de comunicação^8^.

Durante a pandemia COVID-19 a Organização Mundial da Saúde destacou a importância de ações para promover o letramento digital em saúde (LDS) em populações específicas e vulneráveis^8^. A busca pelo LDS que proporcione a obtenção de informação confiável e seu uso adequado aplica-se especialmente aos adolescentes que são usuários constantes da *Internet* e redes sociais^1^. O comportamento saudável nesse período da vida depende mais das decisões individuais e das circunstâncias de vida do que da orientação e ajuda dos pais ou adultos^9^ ^)-(^ ^10^; além disso, aprendizados e hábitos conquistados na adolescência podem ter repercussão por toda a vida adulta.

Norman e Skinner definem o LDS como “a capacidade de buscar, encontrar, compreender e avaliar as informações de saúde de fontes eletrônicas e aplicar os conhecimentos adquiridos para abordar ou resolver um problema de saúde” e propuseram, para a mensuração dessa habilidade, a escala *eHealth Literacy Scale* (eHEALS)^11^. No Brasil, ela foi adaptada transculturalmente^12^ ^)-(^ ^13^, apresentando boas propriedades psicométricas. 

Durante o período pandêmico, em um estudo realizado na Noruega com 2.205 adolescentes de 16 a 19 anos, verificou-se que a higienização das mãos, o distanciamento físico e a limitação do número de contatos sociais foram as medidas mais relatadas pelos adolescentes. Ainda, apontou-se a associação entre o letramento em saúde (LS) e o conhecimento e comportamento relacionados à higiene das mãos, bem como a associação entre o maior LS e menor socialização^2^. O estudo qualitativo conduzido pelos mesmos pesquisadores revela que os adolescentes compreenderam e adotaram as medidas de proteção, como higienização das mãos e distanciamento social, além disso, eles sabiam encontrar informações confiáveis, estavam atentos a elas e faziam o possível para seguir as recomendações para prevenção à COVID-19^14^. 

Apesar da grande importância do LDS como competência essencial para lidar com ameaças à saúde, como a pandemia de COVID-19^2^ ^),(^ ^15^ ^)-(^ ^16^, há evidências limitadas sobre a temática entre adolescentes brasileiros^17^ ^)-(^ ^19^. Assim, o presente estudo teve como objetivo investigar fatores associados ao letramento digital em saúde, ao conhecimento e comportamento para higienização das mãos de adolescentes brasileiros na pandemia de COVID-19.

## Método

### Delineamento e período do estudo

Trata-se de um estudo transversal e analítico, realizado com adolescentes brasileiros das cinco macrorregiões do país, norteado pela diretriz *Strengthening the Reporting of Observational Studies in Epidemiology* (STROBE)^20^ ^)^ e realizado no período de 30 de junho a 08 de agosto de 2021, período em que o Brasil se encontrava em transição entre a terceira fase e quarta fase da pandemia, de forma virtual, por meio da ferramenta *Google Forms*. 

### População e amostra do estudo

A população-alvo foi composta por adolescentes brasileiros entre 15 e 19 anos, com acesso à *Internet.* Foram excluídos aqueles cujos formulários apresentaram falhas no preenchimento. Destaca-se que a faixa etária elegível foi determinada em função do fato de que o Ministério da Saúde segue a definição de adolescência estabelecida pela Organização Mundial da Saúde (OMS): período entre 10 e 19 anos^21^. Além disso, consideramos o fato do eHEALS na versão brasileira ter sido validado em indivíduos a partir de 15 anos^12^. Para o cálculo amostral foi utilizada uma amostra não probabilística do tipo intencional em razão da ausência de uma população referenciada e do interesse do respondente em participar da pesquisa. Para o cálculo da estimativa amostral, considerou-se amostragem aleatória simples, erros tipo I e II iguais a, no máximo, 0,05 e 0,20, respectivamente, correlação linear entre os escores eHEALS e pontuação de conhecimento de higienização das mãos estimada em 0,15 e a presença de cinco variáveis de confundimento nos modelos de análise estatística. Com essas suposições, estimou-se, no mínimo, 387 participantes. A amostra foi constituída por 483 adolescentes, sendo que cinco recusaram-se a participar da pesquisa e cinco foram excluídos por haver falhas no preenchimento dos questionários, resultando em uma amostra final de 473 participantes.

### Variáveis do estudo

#### Desfechos

Avaliação do conhecimento e comportamento em relação à higienização das mãos segundo a versão brasileira do *Questionnaire on Handwashing Knowledge and Behavior* (QHKB-BR) e letramento digital em saúde pelo eHEALS.

#### Variáveis independentes

Variáveis sociodemográficas: sexo (masculino e feminino), região de residência (Norte, Nordeste, Centro-oeste, Sudeste e Sul), idade (em anos), escolaridade (não alfabetizado, ensino fundamental I ou II, ensino médio, graduação), estar cursando ou ter concluído curso técnico ou graduação na área de saúde e referir que um dos responsáveis é profissional da saúde.

### Instrumentos de coleta de dados

Um instrumento de coleta de dados virtual foi desenvolvido na plataforma *Google Forms* pelos autores (por meio do link: https://www.google.com/intl/en-GB/forms/about/), contendo as variáveis relacionadas aos adolescentes referentes à COVID-19, isto é, se a pessoa já teve diagnóstico por COVID-19 (sim ou não); se houve falecimento de familiar por COVID-19 (sim ou não); quais são as situações em que não usa máscaras (reunião com amigos e familiares, prática de esportes, casas noturnas, festas ou bares, domicílio, bares, ar livre e um espaço para apresentar outros locais); se buscava informações sobre a pandemia (sim ou não); quais são os locais de busca de informações sobre a pandemia (Televisão - TV, rádio, jornais, *podcasts*, escolas, *YouTube*, *Twitter* - X, *Snapchat*, *TikTok*, *Instagram* ^*®*^ , *Facebook* ^*®*^ , outras mídias, família, amigos, artigos científicos, universidade ou, ainda, se não busca informações) e o desejo de vacinar (não ou sim; sim, já havia recebido uma dose da vacina; já havia recebido duas doses da vacina ou dose única da vacina).

Como questões abertas, os adolescentes deveriam responder: motivo de não quererem se vacinar (quando coubesse), duas medidas de prevenção da COVID-19 que adotavam e duas medidas que tinham dificuldade de realizar; duas informações que possuíam sobre a vacina contra a COVID-19 e duas informações que gostariam de obter. Na Parte 3 do instrumento, as respostas eram relacionadas ao QHKB-BR^22^ ^)^ e o eHEALS^11^.

O *Questionnaire on Handwashing Knowledge and Behavior* (QHKB) foi desenvolvido na Noruega, para avaliar a higienização das mãos em adolescentes. Contendo seis itens, o questionário foi subdividido em dois domínios: conhecimento (itens 01 a 03) e comportamento (itens 04 a 06). Cada item é medido em uma escala ordinal de cinco níveis (“discordo totalmente”, “discordo”, “nem discordo nem concordo”, “concordo” e “concordo totalmente”)^2^. O QHKB apresentou um alfa de Cronbach alto (0,75 para conhecimento e 0,76 para comportamento)^2^. O QHKB-BR foi adaptado ao contexto do Brasil, e a análise de confiabilidade demonstrou alfa (α) igual a 0,81 e um (ωt) ômega de 0,81. O QHKB-BR apresentou uma consistência interna alta para um instrumento unidimensional, diferentemente da versão original, que foi alta para instrumento bidimensional^22^. A avaliação do conhecimento e comportamento em relação à higienização das mãos ocorreu pelo QHKB-BR, sendo que tanto a pontuação do domínio de conhecimento quanto a de comportamento podiam totalizar de 3 a 15 pontos cada um^22^.

A eHEALS foi uma das primeiras escalas desenvolvidas no Canadá para avaliar as habilidades percebidas dos consumidores no uso da tecnologia da informação, em 664 jovens canadenses com idade de 13 a 21 anos^11^. Ela é composta por oito itens com o objetivo de mensurar o conhecimento, o conforto e as habilidades percebidas dos indivíduos sobre como encontrar, avaliar e aplicar as informações eletrônicas sobre saúde a problemas de saúde^11^. As opções de resposta são dispostas em uma escala Likert de cinco itens, variando de 1 (discordo totalmente) a 5 (concordo totalmente), podendo totalizar de 8 a 40 pontos^11^. Pontuações mais altas no eHEALS pressupõem que o indivíduo possui maiores níveis de LDS^11^. No Brasil, quando aplicada em indivíduos de 15 a 55 anos, apresentou α de Cronbach = 0,89^12^. Quanto à análise fatorial, observou-se um tamanho amostral adequado (KMO = 0,89) com uma matriz de correlação não identitária (teste de esfericidade de Bartlett, p < 0,001) e sem influência da multicolinearidade (determinante = 0,026). A unidimensionalidade do eHEALS foi confirmada pela extração de apenas um fator (valor próprio = 4,43), representando 55,36% da variância total do instrumento^12^. Em indivíduos com idades entre 18 anos e 80 anos, o *eHealth Literacy Scale* - versão brasileira (eHEALS-Br) apresentou excelente consistência interna (α = 0,95 e ω = 0,95), apenas uma dimensão e variância explicada de 81,79^13^.

### Coleta de dados

O recrutamento dos participantes ocorreu por meio da divulgação do formulário digital distribuído por cinco pesquisadores (regiões norte e sudeste) e dois colaboradores (regiões norte e sul), nas mídias sociais e contatos pessoais (*WhatsApp* e *e-mail*). Dispararam-se os convites, que foram multiplicados com uso da técnica “bola de neve”^23^. Os pesquisadores divulgaram as pesquisas em universidades, igrejas, secretarias municipais e estaduais de saúde. 

Assim, uma breve descrição do estudo e seus objetivos foi fornecida aos responsáveis pelos adolescentes menores de 18 anos, de modo que os adultos repassassem as informações aos jovens. Os adolescentes com 18 anos ou mais foram convidados, diretamente, pelos autores. A coleta de dados foi interrompida após o alcance do cálculo amostral com a participação de adolescentes de 26 estados e do Distrito Federal.

### Análise dos dados

Foram ajustados modelos de regressão linear múltipla com resposta normal para explicar as pontuações dos instrumentos QHKB-BR e eHEALS em função das variáveis independentes que se associaram estatisticamente com p < 0,20 na análise bivariada. No modelo final, as associações foram consideradas estatisticamente significativas se p < 0,05. Análises foram feitas com o *software* SPSS 21. Para as perguntas abertas, os conteúdos foram lidos por dois pesquisadores, com supervisão de um terceiro pesquisador, de modo a se analisar se correspondiam às recomendações da OMS^24^. Posteriormente, agrupou-se os dados por similaridades, inserindo-os em uma planilha Excel. Ao final, cada agrupamento de respostas foi lido exaustivamente para comprovar se de fato se relacionam entre si, buscando identificar uma temática que pudesse caracterizar o agrupamento de respostas, realizando-se uma análise descritiva. 

### Aspectos éticos

A pesquisa foi aprovada pelo Comitê de Ética em Pesquisa da Instituição responsável, sob CAAE de número 45521121.1.0000.5411 e parecer número 4.661.979. A aceitação em participar da pesquisa foi registrada por meio do Termo de Consentimento Livre e Esclarecido no formato digital no caso de adolescentes a partir de 18 anos ou de pais/responsáveis pelos menores de 18 anos. Aos participantes menores de 18 anos, além da permissão dos pais, foi solicitada a aceitação do adolescente no Termo de Assentimento Livre e Esclarecido também no formato digital. 

## Resultados

Em relação ao LDS dos adolescentes brasileiros, obteve-se uma média de pontuação de 29,89 pontos (dp±5,30) e mediana de 30,0 (mínimo de 12 e máximo de 40). Para o conhecimento sobre a higienização das mãos foi de 13,1 pontos (dp±1,5) e mediana de 13,0 (mínimo de 7 e máximo 15). Para o comportamento a média foi de 11,1 pontos (dp±2,7) e mediana de 11,0 (mínimo de 3 e máximo 15). Para escore total do conhecimento e comportamento da higienização das mãos, a média foi de 24,3 pontos (dp±3,6), com uma mediana de 24,0 (mínimo de 14 e máximo 30). A caracterização e informações dos adolescentes referentes à COVID-19 estão apresentadas na Tabela 1.

**Tabela 1:** Caracterização sociodemográfica dos adolescentes brasileiros e informações referentes à COVID-19 (n = 473). Brasil, 2023

Variável	Total	
	n*	%^†^
**Sexo**		
Feminino	314	66,4
Masculino	155	32,8
Outro	4	0,8
**Região**		
Sudeste	369	78,0
Nordeste	46	9,7
Norte	26	5,5
Centro-oeste	18	3,8
Sul	14	3,0
**Idade (anos)**		
18-19	267	56,4
15-17	206	43,6
**Escolaridade**		
Ensino médio em andamento	214	45,2
Faculdade em andamento	146	30,9
Ensino médio finalizado	55	11,6
Cursinho preparatório	36	7,6
Ensino fundamental II em andamento	22	4,7
Graduação ou curso técnico na área da saúde	88	18,6
Atividade remunerada	157	33,2
Pais ou responsáveis são da área da saúde	114	24,1
**Escolaridade dos pais/responsáveis**		
Ensino médio	176	37,2
Graduação	121	25,6
Pós-graduação	110	23,3
Ensino fundamental II	40	8,5
Ensino fundamental I	23	4,9
Não alfabetizado	3	0,6
Diagnóstico de COVID-19	81	17,1
Falecimento de familiar por COVID-19	21	4,4
**Situação em que não utiliza máscara**		
Casa	431	91,7
Reuniões familiares	264	55,8
Ambiente ao ar livre	152	32,3
Praticando esporte	94	19,9
Festas	23	4,9
Trabalho	11	2,3
Outros	17	3,6
Busca informações sobre pandemia	379	80,1
**Locais em que buscam as informações**		
Mídias sociais^‡^	443	93,6
Televisão	272	57,4
Jornais	157	33,1
Família	149	31.4
Artigos científicos	115	24,3
Outras mídias^§^	97	20,5
Amigos	95	20,5
Escola	95	20,0
Faculdade	68	14,4
Radio	25	5,28
Sites da internet	11	2,32
**Desejo de receber vacina**	444	93,9

^*^N = Número de total de participantes; ^†^% = Porcentagem; ^‡^ *Instagram* ^*®*^ , *Facebook* ^*®*^ , *Twitter* (X), *TikTok*, *Snapchat*, *podcasts*, *YouTube*; ^§^Outras mídias classificadas pelos adolescentes que não faziam parte do quadro de seleção do questionário

No que se refere às medidas preventivas adotadas pelos participantes, destaca-se que 4,4% (n = 21) não responderam à questão formulada, os demais responderam com uma ou mais medidas preventivas, totalizando 895 medidas de prevenção citadas. Dessas, todas estão de acordo com as medidas de prevenção adotadas pela OMS^24^, exceto a utilização de luvas descartáveis e a utilização de duas máscaras (Tabela 2).

**Tabela 2:** Distribuição das respostas dos adolescentes quanto às medidas preventivas utilizadas e não utilizadas para a prevenção da COVID-19 e às informações que sabem ou gostariam de saber sobre as vacinas para COVID-19. Brasil, 2023

Variável	n*	%^†^
**Medidas preventivas adotadas (N=895)**		
Máscara facial	379	83,8
Higienização das mãos com álcool 70%	252	55,7
Higienização das mãos com água e sabão	91	20,1
Distanciamento social	75	16,6
Isolamento social	67	14,8
Higiene pessoal ou do ambiente	28	6,2
Outras^‡^	03	0,7
**Dificuldades na adoção das medidas preventivas (N=406)**		
Distanciamento social	83	21,7
Máscara facial	73	19,1
Nenhuma dificuldade	62	16,2
Isolamento social	66	17,2
Utilização de álcool ou álcool em gel	46	12,0
Higiene pessoal ou do ambiente	56	14,7
Higienização das mãos	18	4,7
Outros^§^	02	0,5
**Informações que sabem sobre as vacinas (N=402)**		
**Eficácia das vacinas**	102	24,11
Diminui os quadros graves	66	15,60
Efeito colateral	45	10,63
Produção das vacinas	42	9,92
Benefícios das vacinas	28	6,61
Outras^||^	25	5,91
Vacinação em massa	24	5,67
Doses da vacina	23	5,43
Informações negativas	19	4,49
Não sabem	16	3,78
Vacina necessária para o combate à pandemia	12	2,83
**Informações que gostariam de saber (N=319)**		
Eficácia das vacinas	98	29,96
Diferentes tipos de vacinas	60	18,34
Vacinação em adolescentes	50	15,29
Nenhuma	33	10,09
Efeito colateral	29	8,86
Pós-vacina	18	5,50
Doses necessárias	13	3,97
Outras^¶^	10	3,05
Segurança e distribuição	08	2,44

^*^N = Frequência; ^†^% = Porcentagem; ^‡^Ter contato com pessoas que seguem as medidas preventivas, ir ao médico em caso de sintomas, utilização de luvas); ^§^ *Swab* nasal e utilização de luvas; ^||^Segunda dose é um reforço; demora 15 dias para fazer efeito; estão seguindo o calendário vacinal, entre outros; ^¶^Avanço das vacinas, motivos para não vacinar menores de 12 anos, casos pós-vacinas, entre outros

Quanto às dificuldades em aplicar as medidas preventivas, 19,9% (n = 96) não responderam sobre essa questão, os demais responderam com uma ou mais respostas, totalizando 406 dificuldades na adoção de medidas de prevenção. O distanciamento social foi a medida preventiva que os adolescentes assinalaram como a maior dificuldade (21,7%; n = 83) (Tabela 2).

Em relação às informações dos adolescentes sobre as vacinas para prevenção da COVID-19, foram obtidas 402 respostas agrupadas em 11 temáticas principais por um dos pesquisadores, por meio de planilha do Excel. Cada categoria foi nomeada conforme as respostas citadas por cada adolescente, com destaque para as informações sobre a eficácia da vacina (102; 24,11%) e diminuição da gravidade da COVID-19 (66; 15,60%). No tocante às informações que os adolescentes gostariam de obter, 319 responderam a essa questão, totalizando sete temáticas. Da análise das respostas, observou-se que a eficácia da vacina foi o tópico de maior interesse dos adolescentes (29,96%; n = 98) (Tabela 2).

Na Tabela 3, apresentam-se a associação bivariada e regressão linear múltipla ajustada para explicar desfecho QHKB-BR**.** A análise múltipla mostra que o QHKB-BR foi maior entre adolescentes com ensino médio finalizado (b = 2,28 IC95%=(1,13; 3,43); p = 0,000) em comparação àqueles com faculdade, aos que cursavam graduação na área da saúde (b = 1,16 IC95% = (0,19; 2,13); p = 0,019), aos que buscam informações sobre a pandemia (b = 0,97 IC95% = (0,16; 1,78); p = 0,019) e aos que buscam informações via artigos científicos (b = 1,23 IC95% = (0,38; 2,08); p = 0,005). Além disso, a pontuação QHKB-BR foi menor entre homens (b = -0,70 IC95% = (-1,37; -0,02); p = 0,042) e entre adolescentes que referiram não utilizar máscara ao ar livre (b = -0,90 IC95% = (-1,57; -0,23); p = 0,009).

**Tabela 3:** Regressão linear ajustada para explicar desfecho QHKB-BR* (n = 473). Brasil, 2023

Variáveis	Associações bivariadas				Regressão múltipla			
	b^†^	IC^‡^95%		p^§^	b^†^	IC^‡^95%		p^§^
**Sexo**								
Outros^||^	-0,80	-4,35	2,74	0,657	-0,72	-4,68	3,25	0,723
Masculino	-0,83	-1,52	-0,14	0,019	-0,70	-1,37	-0,02	0,042
Feminino^¶^								
**Região**								
Outras regiões	-0,43	-1,21	0,36	0,288				
Sudeste**								
**Idade**								
18-19 anos	0,09	-0,57	0,75	0,786				
15 a 17 anos^††^								
**Escolaridade**								
Cursinho preparatório	-0,34	-1,64	0,96	0,609	0,53	-0,81	1,88	0,438
Ensino fundamental II finalizado	-0,12	-1,72	1,49	0,887	1,21	-0,43	2,86	0,148
Ensino médio em andamento	0,27	-0,48	1,02	0,485	1,13	0,26	2,00	0,011
Ensino médio finalizado	1,77	0,66	2,87	0,002	2,28	1,13	3,43	0,000
Faculdade em andamento^‡‡^								
Cursos de graduação na área da saúde	0,71	-0,13	1,55	0,096	1,16	0,19	2,13	0,019
**Trabalho**								
Sim, fora de casa	-0,43	-1,16	0,30	0,251				
Sim, em *home office*	0,34	-1,13	1,81	0,647				
Trabalho^§§^								
Pais/responsáveis da área da saúde	-0,49	-1,25	0,27	0,208				
**Escolaridade pais ou responsáveis**								
Pós-graduação	2,28	-1,83	6,39	0,277				
Graduação	1,09	-3,02	5,19	0,604				
Ensino médio	1,84	-2,25	5,92	0,378				
Ensino fundamental II	0,98	-3,22	5,18	0,646				
Ensino fundamental I	0,68	-3,63	4,99	0,757				
Não escolarizado^||||^								
**Diagnóstico de COVID-19**	-0,51	-1,38	0,35	0,247				
**Falecimento familiar por COVID-19**	-0,64	-2,22	0,95	0,431				
**Situações em que não utilizam máscaras**								
Reuniões familiares	-0,25	-0,91	0,41	0,462				
Praticando esporte	0,05	-0,77	0,87	0,914				
Festas	-0,25	-1,77	1,27	0,745				
Casa	0,73	-0,46	1,92	0,229				
Trabalho	-1,31	-3,48	0,85	0,235				
Ambiente ao ar livre	-0,93	-1,63	-0,24	0,008	-0,90	-1,57	-0,23	0,009
Outros^¶¶^	1,60	-0,15	3,35	0,074	1,83	0,07	3,60	0,042
**Busca informações sobre pandemia**	1,40	0,58	2,21	0,001	0,97	0,16	1,78	0,019
Locais em que buscam informações								
Artigos científicos	1,49	0,74	2,24	0,000	1,23	0,38	2,08	0,005
Universidades	1,10	0,18	2,03	0,020	0,06	-1,01	1,13	0,913
**Desejo de receber vacina**	0,96	-0,45	2,37	0,181	0,43	-0,95	1,81	0,541

^*^Versão brasileira do *Questionnaire on Handwashing Knowledge and Behavior;* ^*†*^ Intercepto; ^‡^Intervalo de confiança; ^§^Análise bivariada por regressão linear simples/múltipla; ^||^Classificação de gênero de sexo, que não seja masculino ou feminino; ^¶^Referência para sexo; **Referência para regiões no Brasil; ^††^Referência para idade; ^‡‡^Referência para os níveis de escolaridade; ^§§^Referência para trabalho; ^||||^Referência para os níveis de escolaridade dos pais ou responsáveis; ^¶¶^Classificada pelos adolescentes que não faziam parte do quadro de seleção do questionário (familiares próximos vacinados, carro, durante refeições, estar sozinho, pequenos grupos de pessoas)

Na Tabela 4, são apresentadas a associação bivariada e a regressão linear múltipla ajustada para explicar desfecho eHEALS**.** Na análise múltipla, evidencia-se a associação de maiores pontuações de LDS em adolescentes que não utilizaram máscara durante a prática de esportes (b = 1,25 IC95% = (0,23; 2,27); p = 0,017). Houve associação entre LDS e o conhecimento (p = 0,000) e comportamento (p = 0,000) em relação à higienização das mãos. Verificou-se que quanto maior o conhecimento e comportamento em relação à higienização das mãos, maior foi o escore do eHEALS, gerando um aumento no score de 1,28 e 0,40, respectivamente.

**Tabela 4:** Regressão linear ajustada para explicar desfecho eHEALS* (n = 473). Brasil, 2023

Variável	Associações bivariadas				Regressão múltipla			
	b^†^	IC^‡^ 95%		p^§^	b^†^	IC^‡^ 95%		p^§^
**Sexo**								
Masculino	-2,90	-8,12	2,32	0,276				
Feminino	0,05	-0,97	1,07	0,928				
Outros^||^								
**Região**								
Centro-oeste	3,37	0,20	6,53	0,037				
Nordeste	0,97	-1,57	3,50	0,454				
Sul	2,28	-1,14	5,70	0,191				
Sudeste	1,96	-0,14	4,05	0,067				
Norte^¶^								
Outras regiões	-0,638	-1,790	0,514	0,277	-0,24	-1,23	0,76	0,639
**Idade**								
18-19 anos	1,28	0,32	2,24	0,009	0,96	-0,62	2,54	0,235
15 a 17 anos**								
Idade (em anos)	0,42	0,09	0,75	0,013				
**Escolaridade**								
Cursinho preparatório	-1,14	-3,04	0,76	0,241	-0,83	-2,58	0,92	0,352
Ensino fundamental II em andamento	-2,03	-4,36	0,31	0,089	-0,43	-3,04	2,19	0,749
Ensino médio em andamento	-0,73	-1,83	0,36	0,189	0,20	-1,54	1,95	0,820
Ensino médio finalizado	2,07	0,46	3,69	0,012	0,89	-0,63	2,40	0,251
Faculdade^††^								
Cursos na área da saúde	1,14	-0,09	2,36	0,068	0,42	-0,85	1,69	0,514
**Trabalho**								
Não	0,08	-1,00	1,15	0,891				
Sim, fora do domicílio	0,85	-1,30	3,01	0,438				
Sim, em *home office* ^*§§*^								
Pais/responsáveis da área da saúde	-0,20	-1,32	0,91	0,719				
**Escolaridade pais ou responsáveis**								
Pós-graduação	3,62	-2,40	9,64	0,238				
Graduação	2,39	-3,62	8,41	0,435				
Ensino médio	1,93	-4,06	7,92	0,527				
Ensino fundamental II	2,37	-3,79	8,53	0,451				
Ensino fundamental I	3,80	-2,52	10,11	0,239				
Não alfabetizado^||||^								
**Diagnóstico de COVID-19**	-0,24	-1,51	1,03	0,708				
**Falecimento de familiar por COVID-19**	-0,09	-2,42	2,23	0,936				
**Situações em que não utilizavam máscaras**								
Reuniões familiares	0,06	-0,91	1,03	0,902				
Praticando esportes	1,24	0,04	2,43	0,042	1,25	0,23	2,27	0,017
Festas	1,01	-1,22	3,23	0,375				
Casas	-0,35	-2,09	1,39	0,696				
Trabalho	0,83	-2,34	4,01	0,606				
Ambientes ao ar livre	-0,36	-1,39	0,66	0,485				
Outros^¶¶^	0,09	-2,48	2,66	0,945				
**Busca informações sobre pandemia**	1,63	0,43	2,83	0,008	0,53	-0,53	1,59	0,323
**Locais em que buscam as informações**								
Artigos científicos	1,91	0,81	3,02	0,001	0,21	-0,90	1,32	0,707
Universidades	1,93	0,57	3,28	0,005	0,66	-0,73	2,06	0,352
Desejo de receber vacina	-1,17	-3,23	0,89	0,267				
Conhecimento QHKB-BR***	1,69	1,41	1,97	0,000	1,28	0,97	1,59	0,000
Comportamento QHKB-BR***	0,78	0,62	0,95	0,000	0,40	0,22	0,57	0,000

*Escala *eHealth Literacy Scale;* ^*†*^ Intercepto; ^‡^Intervalo de confiança; ^§^Análise bivariada por regressão linear simples/múltipla; ^||^Referência para sexo; ^¶^Referência para regiões no Brasil; ^**^Referência para idade; ^††^Referência para os níveis de escolaridade; ^§§^Referência para trabalho; ^||||^Referência para os níveis de escolaridade dos pais ou responsáveis; ^¶¶^Classificada pelos adolescentes que não faziam parte do quadro de seleção do questionário (familiares próximos vacinados, carro, durante refeições, estar sozinho, pequenos grupos de pessoas); ^***^Versão brasileira do *Questionnaire on Handwashing Knowledge and Behavior*

## Discussão

O destaque do presente estudo foi buscar evidências, por meio de instrumentos validados e perguntas abertas, que pudessem contribuir com a avaliação LDS de adolescentes brasileiros em um momento tão desafiador para todas as populações, fornecendo bases para direcionar ações de políticas públicas no país. Pesquisadores consideram que o LS pode ter algum impacto positivo no controle da disseminação global da COVID-19, porém é necessário que os indivíduos sejam capacitados para adquirir as informações além de entender e saber aplicar os conhecimentos adquiridos^25^.

No presente estudo, a maioria dos adolescentes são do sexo feminino, no entanto, essa variável não se associou com o LDS. Nossos achados estão de acordo com os encontrados no estudo conduzido no Brasil, em que a variável sexo não se associou ao LS^26^. Por outro lado, houve associação entre menores pontuações do QHKB-BR em homens, se comparado com o sexo feminino. Estudiosos acreditam que as mulheres são mais propensas a perceber a COVID-19 como um problema de saúde e acabam aderindo mais às medidas restritivas de política pública. Esses autores pensam também que as diferenças de gênero em atitudes e comportamentos são consideráveis em todos os países^27^.

A maioria dos adolescentes neste estudo citaram a máscara facial como estratégia preventiva adotada durante a COVID-19, porém, ao serem questionados em questão aberta, eles relataram ter dificuldades no uso. Em resposta à prevenção da COVID-19, a OMS já incluía intervenções não farmacêuticas, como uso de máscara, higienização das mãos e distanciamento social, para reduzir a transmissão viral^28^. Uma pesquisa realizada na China^29^ defende que, embora o uso de máscara e a higienização das mãos sejam estratégias utilizadas na prevenção da COVID-19, a formação escolar, a escolaridade da mãe e o local da residência estão associados a um melhor comportamento de uso de máscara e higienização das mãos^29^. Em outra investigação realizada na Turquia^30^ com participantes maiores de 18 anos, apontou-se que os hábitos de higienização das mãos variam por idade e sexo, enquanto os escores de distanciamento social e uso de máscara variam apenas por gênero^30^. 

Embora os escores do QHKB-BR tenham sido satisfatórios para conhecimento e comportamento para a higienização das mãos, quando os adolescentes foram indagados em questão com resposta aberta, eles citaram com mais frequência a higienização com álcool gel em relação à higienização com água e sabão. Uma das suposições seria que os adolescentes entendem que uso de álcool gel seria mais importante que a higienização das mãos com água e sabão. Tais dados sinalizam a importância de ações educativas que promovam o LS dos adolescentes, incluindo as orientações para a higienização das mãos, que esclareçam quando é recomendada a lavagem das mãos com água e sabão e quando esta pode ser substituída pelo uso de álcool em gel. Uma revisão da literatura evidenciou a importância de os profissionais da saúde desempenharem um papel-chave na boa comunicação durante intervenções educativas e ações de educação em saúde com pais/mães/cuidadores, visando à promoção do desenvolvimento infantil^31^. 

Embora a higienização das mãos seja uma recomendação protetora, de baixo custo e amplamente disponível para proteção pessoal e para prevenção epidêmica, nossos achados confirmam que ainda existem desafios na adesão. Assim, encontra-se, na literatura internacional, estratégias interessantes que corroboram com a temática. Um estudo realizado na China^32^ e na Índia^32^, sobre os custos e benefícios dos programas de mudança de comportamento em lavar as mãos, sugere grandes ganhos econômicos relacionados a reduções de surtos epidêmicos por meio de programas de mudança de comportamento nas residências^32^. A pesquisa realizada na Turquia^33^, incluindo alunos do ensino médio, avaliou o comportamento da higienização das mãos e uso de máscara, mostrando a importância de aumentar, por meio de treinamento, a conscientização de pais e crianças em idade escolar sobre medidas preventivas, tais como técnica correta de higienização das mãos (tempo, frequência), seleção de máscara apropriada, assim como modo de uso e descarte^33^.

Quanto às informações que os adolescentes gostariam de ter sobre as vacinas para a prevenção da COVID-19, observou-se que a eficácia da vacina foi o tópico de maior repercussão entre os participantes. Assim percebeu-se o interesse da maioria dos adolescentes em receber a vacina, fato que não sabemos se ocorreu. No entanto, deve-se ter cautela ao analisar esta informação, sendo preciso salientar que a vacinação para o referido grupo ainda estava no início, que grande parte dos participantes eram da região sudeste e, ainda, que 24,1% (n = 114) dos responsáveis eram profissionais da saúde. Destaca-se também que não houve associação entre LDS, QHKB-BR e o desejo de receber a vacina. Em consonância com os nossos achados, a baixa hesitação vacinal foi identificada em um estudo com 526 adolescentes brasileiros de 14 a 19 anos. No estudo, o LS também não influenciou a intenção de não se vacinar, mas sim a percepção da ameaça da COVID-19^19^.

Nesta investigação, verificou-se que a maioria dos adolescentes buscou informações sobre a pandemia em mídias sociais, artigos científicos e na faculdade. No entanto, não houve associação entre o LDS e o local em que os adolescentes buscavam as informações. Por outro lado, houve associação entre maiores escores do QHKB-BR com a busca de informação sobre a pandemia e com a busca de informações em artigos científicos. Em estudo realizado na Austrália com adolescentes de 12 a 17 anos, identificou-se que os recursos *online* acessados para busca não intencional de informações de saúde nas mídias sociais não se mostraram eficazes no LDS para adolescentes, mas sim o uso de estratégias heurísticas ensinadas na escola para pesquisas gerais na *Internet* ^26^. Porém, os autores afirmam que algumas estratégias são inadequadas para o contexto de informação digital em saúde, já que a terminologia dificulta o entendimento ou avaliação das informações de saúde *online* ^26^. Uma pesquisa realizada no Paquistão evidenciou que os adolescentes estão inclinados a usar plataformas digitais para busca de informações sobre saúde^34^.

Nossos achados correspondem às descobertas recentes, mostrando que adolescentes com maior LDS medido pelo eHEALS apresentam maiores escores de conhecimento (p = 0,000) e comportamento em relação à higiene das mãos (p = 0,000). Em consonância com os achados do presente estudo estão os resultados de uma recente pesquisa realizada no Paquistão^34^, incluindo 387 adolescentes, 57,1 % do sexo feminino, 15,8 anos (±1,50), cujo objetivo era avaliar a associação entre comportamento de proteção à saúde e LS para prevenir a propagação da COVID-19. Os autores também encontraram uma associação entre LS e conhecimento em saúde (p=0,023); e entre LS e comportamento em saúde (p < 0,001)^34^. O trabalho conduzido na Polônia^35^ ^)^ corrobora com o nosso estudo, evidenciando que a higienização das mãos está associada com a influência no conhecimento e comportamento dos adolescentes durante o curso da pandemia da COVID-19^35^. Ainda, outro estudo realizado na Noruega^2^ aponta que houve a associação entre o LS e o conhecimento e comportamento de lavar as mãos medido pelo QHKB. 

Além disso, o estudo transversal incluindo 1.322 estudantes universitários japoneses mostrou que altos níveis de LS estão associados a hábitos de vidas saudáveis e, assim, quanto maior a participação dos alunos em aulas relacionadas à educação em saúde, melhor sua competência em acessar e compreender informações no domínio da promoção da saúde^36^. Dessa forma, considera-se relevante promover estratégias contínuas nas escolas, não apenas ações isoladas, para melhorar o LS entre os adolescentes.

Curiosamente, evidenciou-se, nesta pesquisa, a associação entre maiores pontuações de LDS e não utilizar máscara durante a prática de esporte. Nesse sentido, mesmo sabendo que usar máscara era obrigatório, uma possível explicação seria o fato de que o LDS parece ter contribuído para a tomada de decisão dos adolescentes. Uma revisão da literatura sinalizou que o LS pode interferir na tomada de decisão dos adolescentes e que um baixo LS pode levar a decisões e ações de exposição física e mental dessa população, além de consequências que podem comprometer o futuro pessoal, profissional e familiar^18^. 

Os resultados deste estudo fornecem evidências para a compreensão da importância do LDS, que influencia o conhecimento e, consequentemente, o comportamento da higienização das mãos. Para melhorar o LS dos adolescentes, é importante a elaboração de estratégias educativas nas escolas, direcionadas para enfrentar a ameaça de epidemias repentinas de doenças infecciosas graves. O LS de adolescentes tem seis dimensões relacionadas à fase do desenvolvimento, ou seja, seis “D” que precisam ser consideradas no planejamento e execução das ações de saúde: padrões demográficos (adolescentes são particularmente vulneráveis a desigualdades sociais e de saúde), epidemiologia diferencial (algumas doenças e riscos à saúde são altamente específicos de idade para adolescentes), mudança de desenvolvimento (p.ex., biológica, cognitiva, psicológica), dependência (de pais e grupos de pares), cidadania democrática (os adolescentes são atores sociais por direito próprio) e digitalização (a mídia digital é um componente integral de suas vidas diárias)^37^. 

Um estudo realizado na Alemanha com alunos entre 16 e 20 anos, para avaliar um curso de uma plataforma *e-learning*, mostrou que, após a conclusão do curso, os adolescentes atingiram níveis mais elevados de LS, sugerindo que o LDS deve integrar o currículo escolar enquanto parte da estrutura obrigatória para educação digital, com potencial de promover LS em crianças em idade escolar^38^_,_ tal fato poderá impactar positivamente a saúde delas na adolescência e na vida adulta. Do mesmo modo, um editorial publicado na *Lancet*, com autoria de especialistas na área de LS, defende o aproveitamento do potencial das escolas para educar as crianças sobre a saúde e melhorar os processos e estruturas escolares, visando tanto à mudança comportamental como social^39^.

Autores relatam que as condições sociais e ambientais desempenham um importante papel na saúde pública^40^. Ainda, eles afirmam que o LS é considerado direta ou indiretamente ligado a resultados de saúde, por ser um dos Determinantes Sociais da Saúde^41^. Ademais, o baixo LS pode levar a população a ser menos propensa a usar medidas preventivas e serviços voltados a doenças transmissíveis e não transmissíveis, além de menos receptiva às mensagens de educação em saúde^42^ ^)-(^ ^43^.

Algumas limitações da presente pesquisa precisam ser declaradas. A coleta de dados foi concluída em agosto de 2021, quando se iniciou a vacinação nos adolescentes, o que pode ter direcionado as respostas sobre a vacinação. A coleta foi realizada virtualmente, e não puderam participar os adolescentes sem acesso aos recursos eletrônicos (computadores, telefones e *wi-fi*) e aqueles sem habilidade para leitura. Também, não investigamos o nível socioeconômico dos responsáveis e dos adolescentes para associá-lo com o LS nos níveis de prevenção e controle da pandemia de COVID-19.

As descobertas desta pesquisa contribuem para a saúde do adolescente no Brasil produzindo bases para comparação com outros países. Recomenda-se que os profissionais de saúde devam atuar em escolas e universidades em atividades voltadas ao desenvolvimento do LS. Ademais, devem se envolver no desenvolvimento de recursos educativos apropriados e de qualidade, contribuindo para o LDS dos adolescentes. Por fim, ainda é preciso proporcionar atividades educativas sobre a prática de higienização das mãos com água e sabão e com álcool em gel direcionadas aos adolescentes. 

## Conclusão

O desejo de receber a vacina da COVID-19 foi a medida preventiva mais relatada, seguida pela utilização de máscaras faciais, higienização com álcool em gel e, bem menos citada, a higienização das mãos com água e sabão. A eficácia da vacina foi a temática que despertou maior interesse e sobre a qual os adolescentes disseram ter mais informações. 

Existe associação entre o LDS e o conhecimento e comportamento para a higienização das mãos. O LDS influenciou o conhecimento e comportamento dos adolescentes em relação à higiene das mãos e à não utilização de máscara durante a prática de esporte. O conhecimento e comportamento para a higienização das mãos foram maiores entre adolescentes com ensino médio completo em comparação àqueles que cursam ensino superior, aos que frequentam curso da área da saúde, aos que buscam informação sobre a pandemia e àqueles que buscam informações via artigos científicos. 

O estudo sugere que estratégias educativas relacionadas à importância da higienização das mãos e direcionadas aos adolescentes ainda são importantes. Acreditamos que ações educativas devem ser contínuas e iniciadas desde os primeiros anos de vida, com ações intersetoriais que reúnam profissionais da saúde e da educação.

# Translation: Literacidad digital en salud, comportamiento y conocimiento de adolescentes para la higienización de las manos en la pandemia de COVID-19*

**DOI:** 10.1590/1518-8345.7228.4307

**Authors:** Rafaela Aparecida Prata, Juliana Bastoni da Silva, Sidiany Mendes Pimentel, Hélio Rubens de Carvalho Nunes, Marla Andréia Garcia de Avila

**Affiliations:** 1 Universidade Estadual Paulista, Departamento de Enfermagem, Botucatu, SP, Brasil.; 2 Universidade Federal do Tocantins, Departamento de Enfermagem, Palmas, TO, Brasil.

**Keywords:** Alfabetización en Salud, Desinfección de las Manos, Higiene de las Manos, COVID-19, Adolescent, Salud del Adolescente

## Abstract

**Objetivo:**

investigar factores asociados a la literacidad digital en salud, el conocimiento y comportamiento para la higienización de las manos de adolescentes brasileños en la pandemia COVID-19.

**Método:**

estudio transversal con 473 adolescentes de 15 a 19 años. Los datos fueron recogidos virtualmente entre junio y agosto de 2021 mediante un cuestionario de características sociodemográficas, literacidad digital en salud, conocimiento y comportamiento para la higienización de las manos. Las variables fueron ajustadas por modelos de regresión lineal múltiple con respuesta normal.

**Resultados:**

la puntuación media de literacidad digital en salud fue de 29,89 ±5,30; para el conocimiento y comportamiento para la higienización de las manos fue de 13,1 ±1,5 y 11,1±2,7, respectivamente. Mayores puntuaciones de conocimiento y comportamiento para higienización de las manos se asociaron con enseñanza secundaria finalizada, comparados a los que cursan universidad, entre los que asisten a cursos en salud, buscan información sobre la pandemia y en artículos científicos. Mayores puntuaciones de literacidad digital en salud en adolescentes que no utilizaban mascarillas cuando practicaban deporte (p= 0,017). Hubo asociación entre literacidad digital en salud y conocimiento (p = 0,000) y comportamiento (p = 0,000) para higienización de las manos.

**Conclusión:**

existe asociación entre mayores puntuaciones de literacidad digital en salud y el conocimiento y comportamiento para la higienización de las manos.

## Destacados

**(1)** Promedio del alfabetización digital en salud de los adolescentes brasileños fue de 29,89 puntos.

**(2)** Promedio de conocimiento sobre higiene de manos fue de 13,1 puntos y el comportamiento de 11,1.

**(3)** Las puntuaciones de conocimiento y comportamiento fueron menores entre los hombres.

**(4)** Mayor alfabetización digital en salud en adolescentes que no utilizaban mascarillas cuando practicaban deporte.

**(5)** Asociación entre la alfabetización digital en salud y el conocimiento y comportamiento.

## Introducción

Durante la pandemia de COVID-19 (*coronavirus disease*), hubo una avalancha de información que generó incertidumbres y limitaciones en la toma de decisiones por la población; para que las estrategias de protección fueran exitosas, la información relacionada con COVID-19 necesitaba ser comprendida y cumplida^1^ ^)-(^ ^2^.

La práctica de higienización de las manos fue una de las principales estrategias recomendadas para la prevención en la pandemia de COVID-19^3^. Se promovieron campañas nacionales sobre el tema^3^, recomendando la higienización de las manos con agua y jabón^4^ o con el uso de alcohol en su formulación al 70%, recomendado cuando las manos no presentan suciedades visibles al ojo desnudo^4^.

Cada fase de la pandemia presentó diferentes desafíos, variando también entre los países^5^. En Brasil, se distinguieron cinco fases desde marzo de 2020 hasta marzo de 2022^5^. En la primera (de marzo a agosto de 2020), se implementaron estrategias preventivas de higiene de manos, uso de gel antibacterial y de mascarillas en lugares públicos, así como el distanciamiento social. La segunda fase (de septiembre de 2020 a enero de 2021), además de contar con las estrategias preventivas, estuvo marcada por el inicio de una segunda “ola” de la pandemia, en enero de 2021. En la tercera fase (de febrero a junio de 2021), sucedió una crisis generalizada del sistema de salud, circulación de diferentes variantes del virus y vacunación gradual. En la cuarta fase (de julio a noviembre de 2021), se logró un mejor equilibrio del sistema de salud con la efectividad de la vacunación y reducción de la transmisión del virus. En la quinta fase (de diciembre de 2021 a marzo de 2022), apareció una nueva variante (ómicron), y continuó la vacunación^5^. Finalmente, el 5 de mayo de 2023, la Organización Mundial de la Salud (OMS) decretó el fin de la pandemia de COVID-19. Actualmente, en Brasil, el escenario es de circulación de diferentes variantes del virus, cobertura de gran parte de la población por al menos dos dosis de vacunas y evidencias de un tratamiento adecuado^6^.

La infodemia relacionada con COVID-19 fue un desafío presente en todas las fases de la pandemia de COVID-19 y persiste hasta los días actuales^7^. La infodemia se caracterizó por el exceso de información, incluyendo información incorrecta o desactualizada, compartida digitalmente, de persona a persona y por otros medios y canales de comunicación^8^.

Durante la pandemia de COVID-19, la OMS destacó la importancia de acciones para promover la literacidad digital en salud (LDS) en poblaciones específicas y vulnerables^8^. La búsqueda por LDS, que proporcione la obtención de información confiable y su uso adecuado, se aplica especialmente a los adolescentes que son usuarios constantes de internet y redes sociales^1^. El comportamiento saludable en este período de la vida depende más de las decisiones individuales y de las circunstancias de vida que de la orientación y ayuda de los padres o adultos^9^ ^)-(^ ^10^; además, los aprendizajes y hábitos adquiridos en la adolescencia pueden tener repercusiones durante toda la vida adulta.

Norman y Skinner definen LDS como “la capacidad de buscar, encontrar, comprender y evaluar la información de salud de fuentes electrónicas y aplicar los conocimientos adquiridos para abordar o resolver un problema de salud” y propusieron, para la medición de esta habilidad, la escala *eHealth Literacy Scale* (eHEALS)^11^. En Brasil, fue adaptada transculturalmente^12^ ^)-(^ ^13^ presentando buenas propiedades psicométricas. 

Durante el período pandémico, en un estudio en Noruega con 2.205 adolescentes de 16 a 19 años, se verificó que la higienización de las manos, el distanciamiento físico y la limitación del número de contactos sociales fueron las medidas más relatadas por los adolescentes; además, se señaló la asociación entre la literacidad en salud (LS) y el conocimiento y comportamiento relacionados con la higiene de las manos, así como la asociación entre mayor LS y menor socialización^2^. Estudio cualitativo realizado por los mismos investigadores, revela que los adolescentes comprendieron y adoptaron las medidas de protección, como higienización de las manos y distanciamiento social, que sabían encontrar información confiable, que estaban atentos a ella y hacían lo posible por seguir las recomendaciones para la prevención de COVID-19^14^.

A pesar de la gran importancia de la LDS como competencia esencial para manejar amenazas a la salud, como la pandemia de COVID-19^2^ ^),(^ ^15^ ^)-(^ ^16^, hay evidencias limitadas sobre la temática entre adolescentes brasileños^17^ ^)-(^ ^19^. Así, el presente estudio tuvo como objetivo investigar factores asociados a la LDS, el conocimiento y comportamiento para la higienización de las manos de adolescentes brasileños en la pandemia de COVID-19.

## Método

### Delineamento y período del estudio

Se trata de un estudio transversal y analítico, realizado con adolescentes brasileños de las cinco macrorregiones del país, guiado por la directriz *Strengthening the Reporting of Observational Studies in Epidemiology* (STROBE)^20^ y llevado a cabo del 30 de junio al 08 de agosto de 2021 (período en que Brasil estaba en transición entre la tercera y cuarta fase de la pandemia), de forma virtual, mediante la herramienta *Google Forms*.

### Población y muestra del estudio

La población objetivo estuvo compuesta por adolescentes brasileños entre 15 y 19 años, con acceso a internet. Fueron excluidos aquellos cuyos formularios presentaron fallas en el llenado. Se destaca que la franja etaria elegible fue determinada en función del hecho de que el Ministerio de Salud sigue la definición de adolescencia establecida por la OMS: período entre 10 y 19 años^21^. Además, consideramos el hecho de que el eHEALS en la versión brasileña fue validado en individuos a partir de 15 años^12^. Para el cálculo de la muestra se utilizó una muestra no probabilística del tipo intencional debido a la ausencia de una población referenciada y del interés del respondiente en participar en la investigación. Para el cálculo de la estimación muestral, se consideró muestreo aleatorio simple, errores tipo I y II iguales a un máximo de 0,05 y 0,20, respectivamente, correlación lineal entre los puntajes eHEALS y puntuación de conocimiento de higienización de las manos estimada en 0,15 y la presencia de cinco variables de confusión en los modelos de análisis estadístico. Con estas suposiciones, se estimó un mínimo de 387 participantes. La muestra fue constituida por 483 adolescentes, de los cuales cinco se negaron a participar en la investigación y cinco fueron excluidos por haber fallas en el llenado de los cuestionarios, resultando en una muestra final de 473 participantes.

### Variables del estudio

#### Desenlaces

Evaluación del conocimiento y comportamiento en relación a la higienización de las manos según la versión brasileña del *Questionnaire on Handwashing Knowledge and Behavior* (QHKB-BR) y literacidad digital en salud por el eHEALS.

#### Variables independientes

Variables sociodemográficas: sexo (masculino y femenino), región de residencia (Norte, Noreste, Centro-oeste, Sureste y Sur), edad (en años), escolaridad (no alfabetizado, educación primaria o educación primaria ciclo superior, educación secundaria, grado universitario), estar cursando o haber cursado curso técnico o grado en el área de salud y referir que uno de los responsables es profesional de la salud.

### Instrumentos de recolección de datos

Se desarrolló un instrumento de recolección de datos virtual en la plataforma *Google Forms* por los autores (https://www.google.com/intl/en-GB/forms/about/), conteniendo las variables relacionadas con los adolescentes referentes a COVID-19: diagnóstico por COVID-19 (sí o no); fallecimiento de un familiar por COVID-19 (sí o no); situación en que no usan mascarillas (reunión con amigos y familiares, práctica de deportes, discotecas, fiestas o bares, domicilio, bares, al aire libre y un espacio para presentar otros lugares); si buscaban información sobre la pandemia (sí o no); lugar de búsqueda de información sobre la pandemia (Televisión (TV), radio, periódicos, *podcasts*, escuelas, *YouTube*, *Twitter* (X), *Snapchat*, *TikTok*, *Instagram* ^*®*^ , *Facebook* ^*®*^ , otros medios, familia, amigos, artículos científicos, universidad, no busca información) y el deseo de vacunarse (no o sí; sí, ya había recibido una dosis de la vacuna; ya había recibido dos dosis de la vacuna o dosis única de la vacuna).

Como preguntas abiertas, los adolescentes debían responder: motivo de no querer vacunarse (cuando correspondiera), dos medidas de prevención de COVID-19 que adoptaban y dos medidas que tenían dificultad para realizar; dos informaciones que poseían sobre la vacuna contra COVID-19 y dos informaciones que les gustaría obtener.

En la Parte 3 del instrumento, las respuestas estaban relacionadas con el QHKB-BR^22^ y el eHEALS^11^.

El *Questionnaire on Handwashing Knowledge and Behavior* (QHKB) fue desarrollado en Noruega, para evaluar la higienización de las manos en adolescentes conteniendo seis ítems, divididos en dos dominios: conocimiento (ítems 01 a 03) y comportamiento (ítems 04 a 06). Cada ítem se mide en una escala ordinal de cinco niveles (“discrepo totalmente”, “discrepo”, “ni discrepo ni estoy de acuerdo”, “estoy de acuerdo” y “estoy totalmente de acuerdo”)^2^. El QHKB mostró un alfa de Cronbach alto (0,75 para conocimiento y 0,76 para comportamiento)^2^. El QHKB-BR fue adaptado al contexto de Brasil y el análisis de confiabilidad demostró alfa (α) igual a 0,81 y un omega (ωt) de 0,81. El QHKB-BR mostró una consistencia interna alta para un instrumento unidimensional, a diferencia de la versión original, que fue alta para instrumento bidimensional^22^. La evaluación del conocimiento y comportamiento en relación a la higienización de las manos se realizó por el QHKB-BR, siendo que tanto la puntuación del dominio de conocimiento como la de comportamiento podían totalizar de 3 a 15 puntos cada uno^22^.

La eHEALS fue una de las primeras escalas desarrolladas en Canadá para evaluar las habilidades percibidas de los consumidores en el uso de la tecnología de la información, en 664 jóvenes canadienses con edades de 13 a 21 años^11^. Está compuesta por ocho ítems, destinados a medir el conocimiento, la comodidad y las habilidades percibidas de los individuos para encontrar, evaluar y aplicar información de salud electrónica a problemas de salud^11^. Las opciones de respuesta están dispuestas en una escala Likert de cinco ítems, variando de 1 (discrepo totalmente) a 5 (estoy totalmente de acuerdo), pudiendo totalizar de 8 a 40 puntos^11^. Puntuaciones más altas en el eHEALS presuponen que el individuo posee mayores niveles de LDS^11^. En Brasil, cuando aplicada en individuos de 15 a 55 años, presentó α de Cronbach = 0,89^12^. En el análisis factorial, se observó un tamaño muestral adecuado (KMO = 0,89) con una matriz de correlación no identitaria (prueba de esfericidad de Bartlett, p < 0,001) y sin influencia de la multicolinearidad (determinante = 0,026). La unidimensionalidad del eHEALS fue confirmada por la extracción de solo un factor (valor propio = 4,43), representando 55,36% de la varianza total del instrumento^12^. En individuos con edades entre 18 y 80 años, la eHealth *Literacy Scale* - versión brasileña (eHEALS-Br) presentó excelente consistencia interna (α = 0,95 y ω = 0,95), solo una dimensión y varianza explicada de 81,79^13^.

### Recolección de datos

El reclutamiento de los participantes se realizó mediante la divulgación del formulario digital distribuido por cinco investigadores (regiones norte y sureste) y dos colaboradores (regiones norte y sur), por medio de redes sociales y contactos personales (*WhatsApp* y correo electrónico). Se utilizaron invitaciones que se multiplicaron empleando la técnica de “bola de nieve”^23^. Los investigadores promocionaron el estudio en universidades, iglesias y secretarías municipales y estatales de salud. Se proporcionó una breve descripción del estudio y sus objetivos a los responsables de los adolescentes menores de 18 años, para que estos adultos transmitieran la información a los jóvenes. Los adolescentes de 18 años o más fueron invitados directamente por los autores. La recolección de datos se detuvo después de alcanzar el cálculo muestral necesario con la participación de adolescentes de 26 estados y el Distrito Federal.

### Análisis de los datos

Se ajustaron modelos de regresión lineal múltiple con respuesta normal para explicar las puntuaciones de los instrumentos QHKB-BR y eHEALS en función de las variables independientes que se asociaron estadísticamente con p < 0,20 en el análisis bivariado. En el modelo final, las asociaciones se consideraron estadísticamente significativas si p < 0,05. Los análisis se realizaron utilizando el *software* SPSS 21. Para las preguntas abiertas, los contenidos fueron revisados por dos investigadores, bajo la supervisión de un tercer investigador, y se analizó si correspondían a las recomendaciones de la OMS^24^. Posteriormente, se agruparon por similitudes y se trasladaron a una hoja de cálculo de *Excel*. Al final, cada agrupación de respuestas fue examinada exhaustivamente para confirmar si de hecho estaban relacionadas entre sí, se identificó una temática que pudiera caracterizar el agrupamiento de respuestas y se realizó un análisis descriptivo. 

### Aspectos éticos

La investigación fue aprobada por el Comité de Ética en Investigación de la institución responsable, bajo el CAAE número 45521121.1.0000.5411 y el dictamen número 4.661.979. La aceptación para participar en la investigación fue registrada mediante el Término de Consentimiento Informado en formato digital en el caso de los adolescentes a partir de 18 años o de los padres/responsables por los menores de 18 años. A los participantes menores de 18 años, además del permiso de los padres, se solicitó la aceptación del adolescente en el Termo de Asentimiento Informado, también en formato digital.

## Resultados

En relación con la LDS de los adolescentes brasileños, se obtuvo una puntuación media de 29,89 puntos (de±5,30) y una mediana de 30,0 (mínimo de 12 y máximo de 40). Para el conocimiento sobre la higienización de las manos fue de 13,1 puntos (de±1,5) y mediana de 13,0 (mínimo de 7 y máximo de 15). Para el comportamiento, la media fue de 11,1 puntos (de±2,7) y mediana de 11,0 (mínimo de 3 y máximo de 15). Para el puntaje total del conocimiento y comportamiento de la higienización de las manos, la media fue de 24,3 puntos (de±3,6), con una mediana de 24,0 (mínimo de 14 y máximo de 30). La caracterización e información de los adolescentes referente a COVID-19 se presentan en la Tabla 1.

**Tabla 1:** Caracterización sociodemográfica de los adolescentes brasileños e información referente a COVID-19 (n = 473). Brasil, 2023

Variable	Total	
	n*	%^†^
**Sexo**		
Femenino	314	66,4
Masculino	155	32,8
Otro	4	0,8
**Región**		
Sureste	369	78,0
Noreste	46	9,7
Norte	26	5,5
Centro-oeste	18	3,8
Sur	14	3,0
**Edad (años)**		
18-19	267	56,4
15-17	206	43,6
**Escolaridad**		
Educación secundaria en curso	214	45,2
Facultad en curso	146	30,9
Educación secundaria finalizada	55	11,6
Curso preparatorio	36	7,6
Educación primaria en curso (ciclo superior)	22	4,7
Graduación o curso técnico en el área de salud	88	18,6
Actividade remunerada	157	33,2
Padres o responsables son del área de salud	114	24,1
**Escolaridad de los padres/responsables**		
Educación secundaria	176	37,2
Graduación	121	25,6
Posgrado	110	23,3
Educación primaria (ciclo superior)	40	8,5
Educación primaria	23	4,9
No alfabetizado	3	0,6
Diagnóstico de COVID-19	81	17,1
Fallecimiento de familiar por COVID-19	21	4,4
**Situación en que no utiliza mascarilla**		
Casa	431	91,7
Reuniones familiares	264	55,8
Ambiente al aire libre	152	32,3
Practicando deporte	94	19,9
Fiestas	23	4,9
Trabajo	11	2,3
Otras	17	3,6
Busca información sobre pandemia	379	80,1
**Lugares donde buscan la información**		
Medios sociales^‡^	443	93,6
Televisión	272	57,4
Periódicos	157	33,1
Familia	149	31.4
Artículos científicos	115	24,3
Otros medios^§^	97	20,5
Amigos	95	20,5
Escuela	95	20,0
Facultad	68	14,4
Radio	25	5,28
Sitios de la internet	11	2,32
**Deseo de recibir vacina**	444	93,9

^*^N = Número total de participantes; ^†^% = Porcentaje; ^‡^ *Instagram* ^*®*^ , *Facebook* ^*®*^ , *Twitter* (X), *TikTok*, *Snapchat*, *podcasts*, *YouTube*; ^§^Otros medios clasificados por los adolescentes que no formaban parte del cuadro de selección del cuestionario

En lo que respecta a las medidas preventivas adoptadas por los participantes, se destaca que el 4,4% (n = 21) no respondió a la pregunta formulada, y los demás respondieron con una o más medidas preventivas, totalizando 895 medidas de prevención mencionadas. De estas, todas están de acuerdo con las medidas de prevención adoptadas por la OMS^24^, excepto el uso de guantes desechables y el uso de dos mascarillas (Tabla 2).

**Tabla 2:** Distribución de las respuestas de los adolescentes sobre las medidas preventivas utilizadas y no utilizadas para la prevención de COVID-19 y sobre la información que conocen o desearían conocer sobre las vacunas para COVID-19. Brasil, 2023

Variable	n*	%^†^
**Medidas preventivas adoptadas (N=895)**		
Mascarilla	379	83,8
Higienización de las manos con alcohol al 70%	252	55,7
Higienización de las manos con agua y jabón	91	20,1
Distanciamiento social	75	16,6
Aislamiento social	67	14,8
Higiene personal o del ambiente	28	6,2
Otras^‡^	03	0,7
**Dificultades en la adopción de las medidas preventivas (N=406)**		
Distanciamiento social	83	21,7
Mascarilla	73	19,1
Ninguna dificultad	62	16,2
Aislamiento social	66	17,2
Utilización de alcohol o gel antibacterial	46	12,0
Higiene personal o del ambiente	56	14,7
Higienización de las manos	18	4,7
Otras^§^	02	0,5
**Información que conocen sobre las vacunas (N=402)**		
Eficacia de las vacunas	102	24,11
Reduce los cuadros graves	66	15,60
Efecto secundario	45	10,63
Producción de las vacunas	42	9,92
Beneficios de las vacunas	28	6,61
Otras^||^	25	5,91
Vacunación masiva	24	5,67
Dosis de la vacuna	23	5,43
Información negativa	19	4,49
No saben	16	3,78
Vacuna necesaria para combatir la pandemia	12	2,83
**Información que desearían conocer (N=319)**		
Eficacia de las vacunas	98	29,96
Diferentes tipos de vacunas	60	18,34
Vacunación en adolescentes	50	15,29
Ninguna	33	10,09
Efecto secundario	29	8,86
Post-vacunación	18	5,50
Dosis necesarias	13	3,97
Otras^¶^	10	3,05
Seguridad y distribución	08	2,44

^*^N = Frecuencia; ^†^% = Porcentaje; ^‡^Tener contacto con personas que siguen las medidas preventivas, ir al médico en caso de síntomas, uso de guantes; ^§^Hisopo nasal y uso de guantes; ^||^La segunda dosis es un refuerzo; tarda 15 días en hacer efecto; están siguiendo el calendario de vacunación, entre otros; ^¶^Avance de las vacunas, razones para no vacunar a menores de 12 años, casos post-vacuna, entre otros

En cuanto a las dificultades para aplicar las medidas preventivas, el 19,9% (n = 96) no respondió sobre esta cuestión, y los demás respondieron con una o más respuestas, totalizando 406 dificultades en la adopción de medidas de prevención. El distanciamiento social fue la medida preventiva que los adolescentes señalaron como la mayor dificultad (21,7%; n = 83) (Tabla 2).

En relación con la información de los adolescentes sobre las vacunas para la prevención de COVID-19, se obtuvieron 402 respuestas, agrupadas en 11 temáticas principales, por uno de los investigadores mediante una hoja de cálculo de *Excel*; cada categoría fue nombrada según las respuestas citadas por cada adolescente, destacando la información sobre la eficacia de la vacuna (102; 24,11%) y la disminución de la gravedad de COVID-19 (66; 15,60%). En cuanto a la información que los adolescentes desearían obtener, 319 respondieron a esta pregunta, totalizando siete temáticas. Del análisis de las respuestas, se observó que la eficacia de la vacuna fue el tema de mayor interés para los adolescentes (29,96%; n = 98) (Tabla 2).

En la Tabla 3 se presentan la asociación bivariada y la regresión lineal múltiple ajustada para explicar el desenlace QHKB-BR. El análisis múltiple muestra que QHKB-BR fue mayor: entre adolescentes con educación secundaria finalizada (b = 2,28 IC95%=(1,13; 3,43); p=0,000) en comparación con los que están en la universidad, entre adolescentes que cursaban grados en el área de salud (b = 1,16 IC95%=(0,19; 2,13); p=0,019), entre quienes buscan información sobre la pandemia (b = 0,97 IC95%=(0,16; 1,78); p=0,019) y entre quienes buscan información por medio de artículos científicos (b = 1,23 IC95%=(0,38; 2,08); p=0,005). Además, la puntuación QHKB-BR fue menor: entre hombres (b = -0,70 IC95%= (-1,37; -0,02); p=0,042) y entre adolescentes que indicaron no usar mascarillas al aire libre (b = -0,90 IC95%= (-1,57; -0,23); p=0,009).

**Tabla 3:** Regresión lineal ajustada para explicar el desenlace QHKB-BR* (n = 473). Brasil, 2023

Variables	Asociaciones bivariadas				Regresión múltiple			
	b^†^	IC^‡^95%		*p* ^§^	b^†^	IC^‡^95%		*p* ^§^
**Sexo**								
Otros^||^	-0,80	-4,35	2,74	0,657	-0,72	-4,68	3,25	0,723
Masculino	-0,83	-1,52	-0,14	0,019	-0,70	-1,37	-0,02	0,042
Femenino^¶^								
**Región**								
Otras regiones	-0,43	-1,21	0,36	0,288				
Sureste**								
**Idade**								
18-19 anos	0,09	-0,57	0,75	0,786				
15 a 17 anos^††^								
**Escolaridad**								
Curso preparatorio	-0,34	-1,64	0,96	0,609	0,53	-0,81	1,88	0,438
Educación Primaria - Ciclo superior finalizado	-0,12	-1,72	1,49	0,887	1,21	-0,43	2,86	0,148
Educación secundaria en curso	0,27	-0,48	1,02	0,485	1,13	0,26	2,00	0,011
Educación secundaria finalizada	1,77	0,66	2,87	0,002	2,28	1,13	3,43	0,000
Universidad en curso^‡‡^								
Cursos de grado en el área de la salud	0,71	-0,13	1,55	0,096	1,16	0,19	2,13	0,019
**Trabajo**								
Sí, fuera de casa	-0,43	-1,16	0,30	0,251				
Sí, en *home office*	0,34	-1,13	1,81	0,647				
Trabajo^§§^								
Padres/responsables del área de salud	-0,49	-1,25	0,27	0,208				
**Escolaridad padres o responsables**								
Posgrado	2,28	-1,83	6,39	0,277				
Grado	1,09	-3,02	5,19	0,604				
Educación secundaria	1,84	-2,25	5,92	0,378				
Educación primaria - ciclo superior	0,98	-3,22	5,18	0,646				
Educación primaria	0,68	-3,63	4,99	0,757				
No escolarizado^||||^								
**Diagnóstico de COVID-19**	-0,51	-1,38	0,35	0,247				
**Fallecimiento de familiar por COVID-19**	-0,64	-2,22	0,95	0,431				
**Situaciones en que no utilizan mascarillas**								
Reuniones familiares	-0,25	-0,91	0,41	0,462				
Practicando deporte	0,05	-0,77	0,87	0,914				
Fiestas	-0,25	-1,77	1,27	0,745				
Casa	0,73	-0,46	1,92	0,229				
Trabajo	-1,31	-3,48	0,85	0,235				
Ambiente al aire libre	-0,93	-1,63	-0,24	0,008	-0,90	-1,57	-0,23	0,009
Otras^¶¶^	1,60	-0,15	3,35	0,074	1,83	0,07	3,60	0,042
Busca información sobre pandemia	1,40	0,58	2,21	0,001	0,97	0,16	1,78	0,019
Lugares donde buscan la información								
Artículos científicos	1,49	0,74	2,24	0,000	1,23	0,38	2,08	0,005
Universidades	1,10	0,18	2,03	0,020	0,06	-1,01	1,13	0,913
**Deseo de recibir vacuna**	0,96	-0,45	2,37	0,181	0,43	-0,95	1,81	0,541

*Versión brasileña del *Questionnaire on Handwashing Knowledge and Behavior*; ^†^Intercepto; ^‡^Intervalo de confianza; ^§^Análisis bivariado por regresión lineal simple/múltiple; ^||^Clasificación de género de sexo, que no sea masculino o femenino; ^¶^Referencia para sexo; ^**^Referencia para regiones en Brasil; ^††^Referencia para edad; ^‡‡^Referencia para los niveles de escolaridad; ^§§^Referencia para trabajo; ^||||^Referencia para los niveles de escolaridad de los padres o responsables; ^¶¶^Clasificada por los adolescentes que no formaban parte del cuadro de selección del cuestionario (familiares próximos vacunados, coche, durante comidas, estar solo, pequeños grupos de personas)

En la Tabla 4 se presentan la asociación bivariada y la regresión lineal múltiple ajustada para explicar el desenlace eHEALS. En el análisis múltiple se evidencia la asociación de mayores puntuaciones de LDS en adolescentes que no utilizaron mascarilla durante la práctica de deportes (b = 1,25 IC95%= (0,23; 2,27); p=0,017). Hubo asociación entre LDS y el conocimiento (p =0,000) y comportamiento (p =0,000) en relación con la higienización de las manos. Se verificó que, cuanto mayor el conocimiento y comportamiento en relación con la higienización de las manos, mayor fue el puntaje del eHEALS, generando un aumento en el puntaje de 1,28 y 0,40, respectivamente.

**Tabla 4:** Regresión lineal ajustada para explicar el desenlace eHEALS* (n = 473). Brasil, 2023

Variable	Asociaciones bivariadas				Regresión múltiple			
	b^†^	IC^‡^ 95%		p^§^	b^†^	IC^‡^ 95%		p^§^
**Sexo**								
Masculino	-2,90	-8,12	2,32	0,276				
Femenino	0,05	-0,97	1,07	0,928				
Otros^||^								
**Región**								
Centro-oeste	3,37	0,20	6,53	0,037				
Noreste	0,97	-1,57	3,50	0,454				
Sur	2,28	-1,14	5,70	0,191				
Sureste	1,96	-0,14	4,05	0,067				
Norte^¶^								
Otras regiones	-0,638	-1,790	0,514	0,277	-0,24	-1,23	0,76	0,639
**Edad**								
18-19 anos	1,28	0,32	2,24	0,009	0,96	-0,62	2,54	0,235
15 a 17 anos**								
Edad (en años)	0,42	0,09	0,75	0,013				
**Escolaridad**								
Curso preparatorio	-1,14	-3,04	0,76	0,241	-0,83	-2,58	0,92	0,352
Educación primaria - ciclo superior en curso	-2,03	-4,36	0,31	0,089	-0,43	-3,04	2,19	0,749
Educación secundaria en curso	-0,73	-1,83	0,36	0,189	0,20	-1,54	1,95	0,820
Educación secundaria finalizada	2,07	0,46	3,69	0,012	0,89	-0,63	2,40	0,251
Facultad^††^								
Cursos en el área de la salud	1,14	-0,09	2,36	0,068	0,42	-0,85	1,69	0,514
**Trabajo**								
No	0,08	-1,00	1,15	0,891				
Sí, fuera de casa	0,85	-1,30	3,01	0,438				
Sí, en *home office* ^*§§*^								
Padres/responsables del área de la salud	-0,20	-1,32	0,91	0,719				
**Escolaridad padres o responsables**								
Posgrado	3,62	-2,40	9,64	0,238				
Grado	2,39	-3,62	8,41	0,435				
Educación secundaria	1,93	-4,06	7,92	0,527				
Educación primaria ciclo superior	2,37	-3,79	8,53	0,451				
Educación primaria	3,80	-2,52	10,11	0,239				
No alfabetizado^||||^								
**Diagnóstico de COVID-19**	-0,24	-1,51	1,03	0,708				
**Fallecimiento de familiar por COVID-19**	-0,09	-2,42	2,23	0,936				
**Situaciones en las que no utilizaban mascarillas**								
Reuniones familiares	0,06	-0,91	1,03	0,902				
Practicando deportes	1,24	0,04	2,43	0,042	1,25	0,23	2,27	0,017
Fiestas	1,01	-1,22	3,23	0,375				
Casas	-0,35	-2,09	1,39	0,696				
Trabajo	0,83	-2,34	4,01	0,606				
Ambientes al aire libre	-0,36	-1,39	0,66	0,485				
Otros^¶¶^	0,09	-2,48	2,66	0,945				
**Busca información sobre la pandemia**	1,63	0,43	2,83	0,008	0,53	-0,53	1,59	0,323
**Lugares donde buscan la información**								
Artículos científicos	1,91	0,81	3,02	0,001	0,21	-0,90	1,32	0,707
Universidades	1,93	0,57	3,28	0,005	0,66	-0,73	2,06	0,352
Deseo de recibir vacuna	-1,17	-3,23	0,89	0,267				
Conocimiento QHKB-BR***	1,69	1,41	1,97	0,000	1,28	0,97	1,59	0,000
Comportamiento QHKB-BR***	0,78	0,62	0,95	0,000	0,40	0,22	0,57	0,000

*Escala *eHealth Literacy Scale*; ^†^Intercepto; ^‡^Intervalo de confianza; ^§^Análisis bivariado por regresión lineal simple/múltiple; ^||^Referencia para sexo; ^¶^Referencia para regiones en Brasil; **Referencia para edad; ^††^Referencia para los niveles de escolaridad; ^§§^Referencia para trabajo; ^||||^Referencia para los niveles de escolaridad de los padres o responsables; ^¶¶^Clasificada por los adolescentes que no formaban parte del cuadro de selección del cuestionario (familiares próximos vacunados, coche, durante comidas, estar solo, pequeños grupos de personas); ***Versión brasileña del *Questionnaire on Handwashing Knowledge and Behavior*

## Discusión

El enfoque de este estudio fue buscar evidencias, mediante instrumentos validados y preguntas abiertas, que pudieran contribuir con la evaluación de LDS en adolescentes brasileños en un momento tan desafiante para todas las poblaciones y proporcionar bases para dirigir acciones de políticas públicas en el país. Los investigadores consideran que la LS puede tener algún impacto positivo en el control de la diseminación global de la COVID-19, sin embargo, es necesario que los individuos estén capacitados para adquirir la información además de entender y saber aplicar los conocimientos adquiridos^25^.

En el presente estudio, la mayoría de los adolescentes son del sexo femenino, sin embargo, esta variable no se asoció con la LDS. Nuestros hallazgos están de acuerdo con los encontrados en el estudio conducido en Brasil, donde la variable sexo no se asoció con la LS^26^. Por otro lado, hubo una asociación entre menores puntuaciones del QHKB-BR en hombres, en comparación con el sexo femenino. Los estudiosos creen que las mujeres son más propensas a percibir la COVID-19 como un problema de salud y terminan adhiriendo más a las medidas restrictivas de política pública; y estos autores también piensan que las diferencias de género en actitudes y comportamientos son considerables en todos los países^27^.

La mayoría de los adolescentes en este estudio citaron la mascarilla como estrategia preventiva adoptada durante la COVID-19, sin embargo, al ser cuestionados en una pregunta abierta, reportaron tener dificultades en su uso. En respuesta a la prevención de la COVID-19, la OMS ya incluía intervenciones no farmacéuticas, como el uso de mascarilla, higienización de las manos y distanciamiento social, para reducir la transmisión viral^28^. Un estudio realizado en China^29^ defiende que, aunque el uso de mascarilla y la higienización de las manos son estrategias utilizadas en la prevención de la COVID-19, la formación escolar, la escolaridad de la madre y el lugar de residencia están asociados a un mejor comportamiento de uso de mascarilla y higienización de las manos^29^. Otra investigación realizada en Turquía^30^ con participantes mayores de 18 años, indicó que los hábitos de higienización de las manos varían por edad y sexo, mientras que los puntajes de distanciamiento social y uso de mascarilla varían solo por género^30^.

Aunque los puntajes del QHKB-BR fueron satisfactorios para conocimiento y comportamiento en relación con la higienización de las manos, cuando los adolescentes fueron cuestionados en una pregunta con respuesta abierta, citaron más frecuentemente la higienización con gel antibacterial que la higienización con agua y jabón. Una de las suposiciones sería que los adolescentes entienden que el uso de gel antibacterial es más importante que la higienización de las manos con agua y jabón. Estos datos señalan la importancia de acciones educativas que promuevan la LS de los adolescentes, incluyendo las orientaciones para la higienización de las manos, que aclaren cuándo es recomendada la lavada de manos con agua y jabón y cuándo esta puede ser sustituida por el uso de gel antibacterial. Una revisión de la literatura evidenció la importancia de que los profesionales de la salud desempeñen un papel clave en la buena comunicación durante intervenciones educativas y acciones de educación en salud con padres/madres/cuidadores, con el objetivo de promover el desarrollo infantil^31^. 

Aunque la higiene de las manos es una recomendación protectora, económica y ampliamente disponible para la protección personal y la prevención epidémica, nuestros hallazgos confirman que todavía existen desafíos en la adhesión. Por lo tanto, en la literatura internacional se encuentran estrategias interesantes que corroboran con la temática. Un estudio realizado en China^32^ y en la India^32^ sobre costos y beneficios de los programas de cambio de comportamiento en el lavado de manos sugiere grandes ganancias económicas relacionadas con la reducción de brotes epidémicos por medio de programas de cambio de comportamiento en los hogares^32^. Una investigación realizada en Turquía^33^, que incluyó a estudiantes de secundaria, evaluó el comportamiento de la higiene de las manos y el uso de mascarillas, mostrando la importancia de aumentar, por intermedio de la capacitación, la conciencia de los padres y los niños en edad escolar sobre medidas preventivas, como la técnica correcta de higiene de manos (tiempo, frecuencia), selección de mascarilla apropiada, así como modo de uso y eliminación^33^.

En cuanto a la información que los adolescentes desearían tener sobre las vacunas para la prevención de la COVID-19, se observó que la eficacia de la vacuna fue el tema de mayor interés entre los participantes. Así, se percibió el interés de la mayoría de los adolescentes en recibir la vacuna, hecho que desconocemos si ocurrió. Sin embargo, se debe tener precaución al analizar esta información, y es necesario señalar que la vacunación para este grupo aún estaba en sus inicios, que gran parte de los participantes eran de la región sudeste, y además, que el 24,1% (n=114) de los responsables eran profesionales de la salud. También se destaca que no hubo asociación entre la LDS y QHKB-BR y el deseo de recibir la vacuna. En consonancia con nuestros hallazgos, la baja vacilación vacunal fue identificada en un estudio con 526 adolescentes brasileños de 14 a 19 años. En el estudio, la LS también no influyó en la intención de no vacunarse, sino más bien en la percepción de la amenaza de la COVID-19^19^.

En esta investigación, se encontró que la mayoría de los adolescentes buscaban información sobre la pandemia en redes sociales, artículos científicos y en la universidad. Sin embargo, no hubo asociación entre la LDS y el lugar donde los adolescentes buscaban la información. Por otro lado, hubo asociación entre mayores puntuaciones en el QHKB-BR y la búsqueda de información sobre la pandemia y con la búsqueda de información en artículos científicos. En un estudio realizado en Australia con adolescentes de 12 a 17 años, se identificó que los recursos en línea utilizados para la búsqueda no intencional de información de salud en las redes sociales no fueron efectivos en la LDS para adolescentes, sino que el uso de estrategias heurísticas enseñadas en la escuela para investigaciones generales en internet^26^. Sin embargo, los autores afirman que algunas estrategias son inadecuadas para el contexto de información digital en salud, ya que la terminología dificulta la comprensión o evaluación de la información de salud en línea^26^. Una investigación realizada en Pakistán evidenció que los adolescentes tienden a utilizar plataformas digitales para buscar información sobre salud^34^.

Nuestros hallazgos corresponden con descubrimientos recientes, mostrando que los adolescentes con una mayor LDS medido por eHEALS presentan mayores puntuaciones de conocimiento (p = 0,000) y comportamiento en relación con la higiene de manos (p = 0,000). En consonancia con los hallazgos del presente estudio, se encuentran los resultados de una investigación reciente realizada en Pakistán^34^ que incluyó a 387 adolescentes, 57,1 % de sexo femenino, con una edad promedio de 15,8 años (±1,50), cuyo objetivo era evaluar la asociación entre el comportamiento de protección de la salud y la LS para prevenir la propagación de la COVID-19. Los autores también encontraron una asociación entre la LS y el conocimiento en salud (p=0,023); y entre la LS y el comportamiento en salud (p < 0,001)^34^. Un trabajo realizado en Polonia^35^ respalda nuestro estudio, evidenciando que la higiene de manos está asociada con la influencia en el conocimiento y el comportamiento de los adolescentes durante el transcurso de la pandemia de la COVID-19^35^. Un estudio realizado en Noruega^2^ señala que hubo asociación entre la LS y el conocimiento y comportamiento de lavado de manos medido por el QHKB. 

Un estudio transversal que incluyó a 1.322 estudiantes universitarios japoneses mostró que niveles altos de LS están asociados con hábitos de vida saludables, y cuanto mayor es la participación de los alumnos en clases relacionadas con la educación en salud, mejor es su competencia para acceder y comprender información de salud en el ámbito de la promoción de la salud^36^. Por lo tanto, se considera relevante promover estrategias continuas en las escuelas, no solo acciones aisladas, para mejorar la LS entre los adolescentes.

Curiosamente, en esta investigación se evidenció la asociación entre mayores puntuaciones de LDS y no usar mascarilla durante la práctica de deporte. En este sentido, aunque se sabía que el uso de mascarilla era obligatorio, una posible explicación sería que la LDS parece haber contribuido a la toma de decisiones de los adolescentes. Una revisión de la literatura indicó que la LS puede interferir en la toma de decisiones de los adolescentes y que una baja LS puede llevar a decisiones y acciones de exposición física y mental en esta población, además de consecuencias que pueden comprometer el futuro personal, profesional y familiar^18^. 

Los resultados de este estudio proporcionan evidencia para comprender la importancia de la LDS, que influye en el conocimiento y, consecuentemente, en el comportamiento de la higiene de manos. Para mejorar la LS de los adolescentes, es importante desarrollar estrategias educativas en las escuelas dirigidas a enfrentar la amenaza de epidemias repentinas de enfermedades infecciosas graves. La LS de los adolescentes tiene seis dimensiones relacionadas con esta etapa del desarrollo (es decir, seis “D”), que deben tenerse en cuenta en la planificación y ejecución de acciones de salud: patrones demográficos (los adolescentes son particularmente vulnerables a desigualdades sociales y de salud), epidemiología diferencial (algunas enfermedades y riesgos para la salud son altamente específicos de la edad adolescente), cambio de desarrollo (por ejemplo, biológico, cognitivo, psicológico), dependencia (de padres y grupos de pares), ciudadanía democrática (los adolescentes son actores sociales por derecho propio) y digitalización (los medios digitales son un componente integral de sus vidas diarias)^37^. Un estudio realizado en Alemania con estudiantes de entre 16 y 20 años, para evaluar un curso de una plataforma de *e-learning*, mostró que, después de completar el curso, los adolescentes alcanzaron niveles más altos de LS, lo que sugiere que la LDS debería integrarse en el plan de estudios escolares como parte de la estructura obligatoria para la educación digital, con el potencial de promover la LS en niños en edad escolar^38^, lo que podría tener un impacto positivo en su salud durante la adolescencia y la vida adulta. Del mismo modo, un editorial publicado en *The Lancet*, escrito por expertos en el área de LS, aboga por aprovechar el potencial de las escuelas para educar a los niños sobre la salud y mejorar los procesos y estructuras escolares, con el objetivo tanto de cambio de comportamiento como social^39^.

Los autores señalan que las condiciones sociales y ambientales juegan un papel importante en la salud pública^40^. Además, afirman que la LS se considera directa o indirectamente relacionado con los resultados de salud, al ser uno de los Determinantes Sociales de la Salud^41^. Además, la baja LS puede llevar a la población a ser menos propensa a utilizar medidas preventivas y servicios dirigidos a enfermedades transmisibles y no transmisibles, además de ser menos receptiva a los mensajes de educación en salud^42^ ^)-(^ ^43^.

Algunas limitaciones de la presente investigación deben ser mencionadas. La recolección de datos se completó en agosto de 2021, cuando comenzó la vacunación en adolescentes, lo que podría haber influido en las respuestas sobre la vacunación. Esta recolección se realizó de forma virtual, por lo que los adolescentes sin acceso a recursos electrónicos (computadoras, teléfonos y Wi-Fi) y aquellos sin habilidades de lectura no pudieron participar. Además, no investigamos el nivel socioeconómico de los responsables y los adolescentes para asociarlo con la LS en los niveles de prevención y control de la pandemia de COVID-19.

Los hallazgos de esta investigación contribuyen a la salud del adolescente en Brasil al proporcionar bases para la comparación con otros países. Se recomienda que los profesionales de la salud trabajen en escuelas y universidades en actividades dirigidas al desarrollo de la LS. Además, deben involucrarse en el desarrollo de recursos educativos apropiados y de calidad, contribuyendo a la LDS de los adolescentes. Por último, aún es necesario proporcionar actividades educativas sobre la práctica de higiene de manos con agua y jabón y con alcohol en gel dirigidas a los adolescentes. 

## Conclusión

El deseo de recibir la vacuna contra la COVID-19 fue la medida preventiva más mencionada, seguida por el uso de mascarillas, la higienización con gel antibacterial; y mucho menos mencionada, la higienización de manos con agua y jabón. La eficacia de la vacuna fue el tema que despertó mayor interés y sobre el cual los adolescentes dijeron tener más información.

Existe una asociación entre la LDS y el conocimiento y comportamiento para la higienización de manos. La LDS influyó en el conocimiento y comportamiento de los adolescentes en relación con la higiene de manos y la no utilización de mascarillas durante la práctica de deporte. El conocimiento y comportamiento para la higienización de manos fueron mayores entre los adolescentes con educación secundaria completa en comparación con aquellos que cursan educación superior, entre los que asisten a cursos en el área de la salud, entre los que buscan información sobre la pandemia y entre los que buscan información por medio de artículos científicos.

El estudio sugiere que las estrategias educativas relacionadas con la importancia de la higienización de manos y dirigidas a los adolescentes siguen siendo importantes. Creemos que las acciones educativas deben ser continuas y comenzar desde los primeros años de vida, con acciones intersectoriales que reúnan a profesionales de la salud y de la educación.
